# *DNM2*–Charcot–Marie–Tooth neuropathy stems from disrupted Schwann cell function and shows limited therapeutic reversibility

**DOI:** 10.1093/braincomms/fcag324

**Published:** 2026-09-01

**Authors:** Marie Goret, Thomas Arbogast, Jocelyn Laporte

**Affiliations:** Institute of Genetics and Molecular and Cellular Biology (IGBMC), INSERM U1258, CNRS UMR7104, University of Strasbourg, Illkirch 67404, Strasbourg, France; Institute of Genetics and Molecular and Cellular Biology (IGBMC), INSERM U1258, CNRS UMR7104, University of Strasbourg, Illkirch 67404, Strasbourg, France; Institute of Genetics and Molecular and Cellular Biology (IGBMC), INSERM U1258, CNRS UMR7104, University of Strasbourg, Illkirch 67404, Strasbourg, France

**Keywords:** hereditary motor and sensory neuropathy, Charcot–Marie–Tooth neuropathy, dynamin, gene therapy, adeno-associated virus

## Abstract

Dominant loss-of-function mutations in *DNM2* cause Charcot–Marie–Tooth neuropathy characterized by sensory and motor deficits associated with myelin and/or axonal abnormalities and muscle atrophy. Increasing DNM2 activity from embryogenesis has been reported to ameliorate neuromuscular phenotypes in the *Dnm2^K562E/+^* Charcot–Marie–Tooth mouse; however, this model displays predominantly muscle pathology and limited nerve involvement, precluding rigorous evaluation of neuropathic mechanisms and potential therapies. Here, we performed comprehensive behavioural, electrophysiological, histological and molecular analyses to characterize the *Dnm2^K562E/^*^SC−^ mouse, which combines systemic heterozygosity for the common K562E mutation together with Schwann cell-specific deletion of wild-type *Dnm2*. This model faithfully reproduces key clinical and pathological features of *DNM2*–Charcot–Marie–Tooth, including motor deficits, reduced general force and coordination, and severe sensory and motor conduction deficits associated with axonal loss, demyelination and inflammation. Mechanistically, we delineate a coherent pathological sequence that explains the profound functional deficits. In particular, a downregulation of the transcription factor EGR2, a master regulator of myelin gene expression, and of the myelin protein MPZ correlates with demyelination. To evaluate the therapeutic potential of DNM2 supplementation, post-symptomatic intrathecal delivery of AAV9-DNM2 driven by the Schwann cell-specific MPZ promoter was performed at 4 weeks. Although DNM2 expression increased in peripheral nerves (∼1.9-fold), no significant improvements were observed across behavioural, electrophysiological, structural or molecular parameters. Together, these findings establish the *Dnm2^K562E/^*^SC−^ mouse as a robust preclinical model, recapitulating key features of *DNM2*–Charcot–Marie–Tooth, and provide crucial insight into the biological and temporal constraints that must guide future therapeutic strategies for *DNM2*–Charcot–Marie–Tooth.

## Teaser

Goret *et al*. characterize a mouse model, *Dnm2^K562E/^*^SC−^, combining a heterozygous K562E dynamin-2 mutation with Schwann cell-specific deletion, recapitulating key Charcot–Marie–Tooth disease features including motor deficits, nerve conduction defects, axonal loss, demyelination, inflammation and muscle atrophy. Intrathecal adeno-associated virus delivery of dynamin-2 after disease onset showed no disease reversion.

## Graphical Abstract

**Graphical Abstract:**
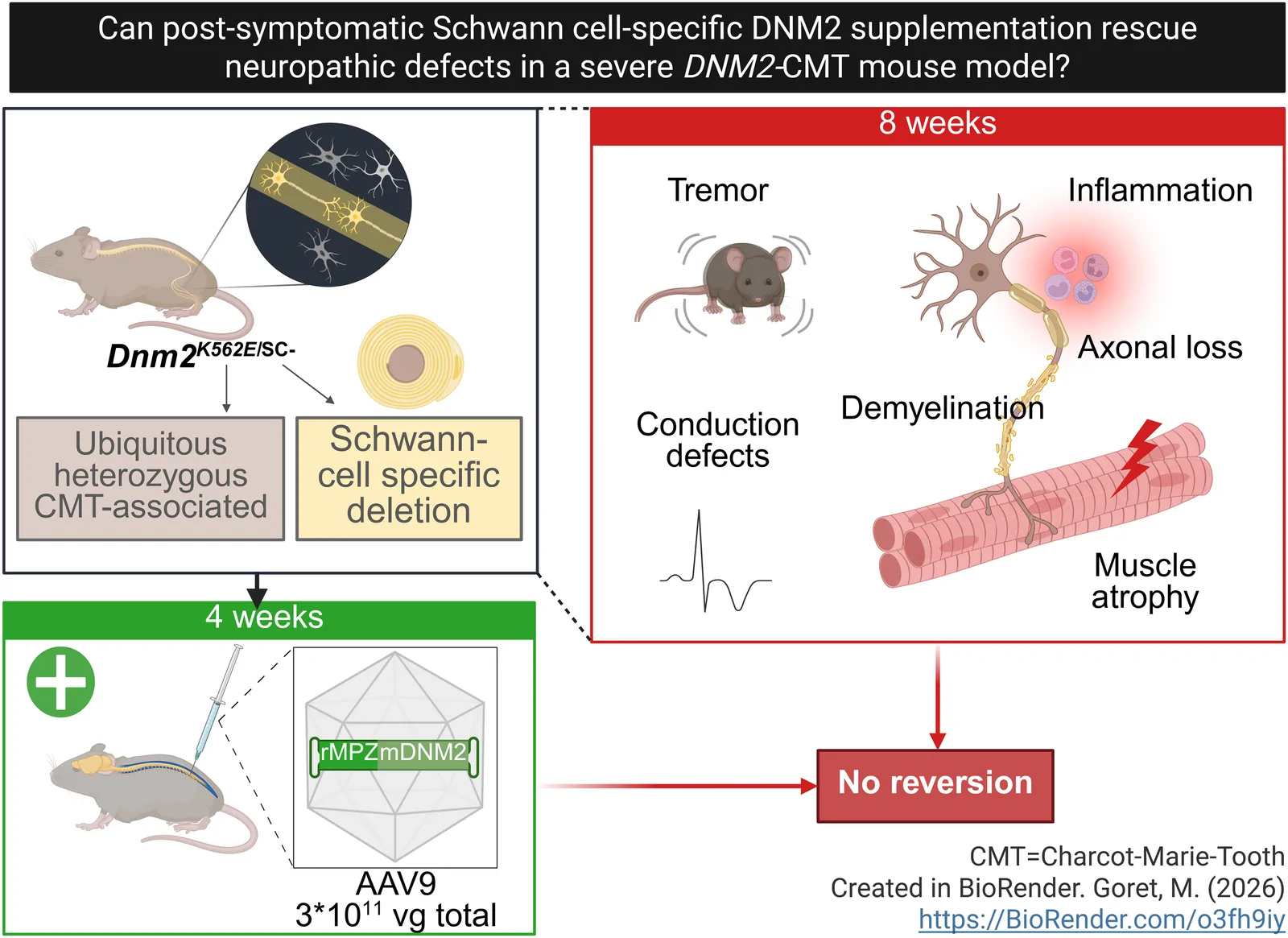

## Introduction

Charcot–Marie–Tooth disease (CMT) is the most common inherited peripheral neuropathy, with more than 100 causative genes identified across demyelinating and axonal forms of the disorder.^1^ Among these, *DNM2* encodes dynamin 2, a large GTPase involved in membrane trafficking and cytoskeletal dynamics.^2-4^ Dominant *DNM2* mutations cause both intermediate (CMT-DIB; MIM#606482)^5,6^ and axonal (CMT2M)^7^ forms of CMT. Patients with *DNM2*-related CMT typically present with slowed nerve conduction velocities, impaired proprioception, decreased reflexes, progressive distal weakness and atrophy, gait disturbances, and skeletal deformities.^8^ Additional features such as ptosis, ophthalmoparesis, cataracts or neutropenia may occur.^9-11^ Nerve biopsies commonly reveal loss of large myelinated fibres, clusters of regenerating axons, focal myelin thickening, increased g-ratio, occasional onion bulb formation and few fibres with Schmidt–Lanterman incisures.^10-13^ Currently, no therapies are available,^14,15^ underscoring the need for mechanistic and therapeutic studies.

The mechanisms underlying *DNM2*-related CMT are not fully understood. Several studies highlighted that *DNM2*–CMT most probably arises from a DNM2 dominant-negative loss-of-function mechanism. Mutations lead to decreased GTPase activity,^16,17^ and complete *Dnm2* deletion in Schwann cells (SCs) resulted in severely impaired axonal sorting and myelination onset leading to a rapidly developing peripheral neuropathy.^18^ However, the *Dnm2^K562E/+^* mouse, carrying the most frequent patient mutation,^19^ exhibits motor and muscle abnormalities but only subtle nerve pathology.^20-22^ Consequently, this model offers limited insight into Schwann cell-specific mechanisms and hinders the evaluation of nerve-targeted therapeutic strategies. Previous preclinical studies have suggested that enhancing DNM2 activity could compensate for the loss-of-function mechanism underlying CMT. Indeed, genetic crosses that increased DNM2 activity from embryogenesis, either by introducing the hyperactive DNM2-S619L variant or reducing BIN1, a negative regulator of DNM2, rescued neuromuscular phenotypes in *Dnm2^K562E/+^* mice.^20,22^ However, these proof-of-concept strategies relied on genetic crosses that increased DNM2 activity ubiquitously from embryogenesis, limiting their translational potential. The feasibility of a postnatal, gene-supplementation-based approach to restore DNM2 function in peripheral nerves has not yet been explored.

To address these gaps, we performed here a detailed phenotypic characterization of the *Dnm2^K562E/^*^SC−^ mouse model, which combines systemic expression of the K562E mutant allele with Schwann cell (SC)-specific deletion of wild-type (WT) *Dnm2.*^21^ This model exhibits a severe demyelinating neuropathy and provides a platform to dissect Schwann cell-autonomous disease mechanisms. We further evaluated whether targeted DNM2 augmentation after symptom onset could reverse neuropathic pathology by delivering AAV9-DNM2 under the Schwann cell-specific MPZ promoter via intrathecal injection, followed by comprehensive behavioural, electrophysiological, histological and molecular assessments. *Dnm2^K562E/^*^SC−^ mice exhibited tremor, severe coordination deficits, conduction impairments, axonal loss and demyelination associated with reduced MPZ and EGR2 expression and increased inflammation, thereby recapitulating a demyelinating neuropathy closely resembling that observed in patients. Importantly, DNM2 supplementation at 4 weeks of age failed to reverse these defects, indicating that DNM2 activity is required during early postnatal Schwann cell maturation and suggesting that late Schwann cell-specific DNM2 augmentation is insufficient to repair established pathology.

## Materials and methods

### Ethical approval

All procedures involving animals were performed in accordance with French and European Union legislation governing animal experimentation and received approval from the Com'Eth IGBMC-ICS institutional ethics committee (Illkirch, France) under authorization reference APAFIS #43721-202304241436921.

### Mouse models

*Dnm2^K562E/^*^SC−^ mice were generated through a two-step breeding strategy. First, MPZ-Cre^+^ mice (strain #017927, RRID:IMSR_JAX:017927; obtained from Charles River) were crossed with *Dnm2* exon 8 floxed mice (ICS project K546/IR2694).^23^ The resulting MPZ-Cre^+^ *Dnm2^fl/+^* progeny (*Dnm2^+/^*^SC−^) were then crossed with *Dnm2^K562E/+^* mice^20,21^ to produce experimental animals. All mice, including controls, expressed MPZ-Cre, had a mixed genetic background (75% C57BL/6J and 25% C57BL/6N), included both sexes and were analysed at 8 weeks of age. Of note, MPZ-Cre expression initiates between embryonic days 13.5 and 14.^24^

For genotyping, primers ‘6115 Er KE’ and ‘6116 Ef KE’ were used to detect K562E mutation, primers ‘Cre 160’ and ‘Cre 161’ to detect the Cre and ‘4613’ and ‘4614’ for the *Dnm2* exon 8 floxed allele (Supplementary Table 1 Reagents).

Animals were housed under standard ventilated conditions with free access to food and water. The holding room was maintained at 19–22°C with 40–60% relative humidity, under a 12-h light/dark cycle. Breeding mice received SAFE® D03 diet, transitioning to SAFE® D04 post-weaning, while *Dnm2^K562E/^*^SC−^ mice remained on SAFE® D03 supplemented with DietGel® 76A (SKU: 72-07-5022) from 3 weeks.

### AAV vector design and production

Recombinant AAV9 vectors were produced at the Molecular Biology and Virus Facility (IGBMC) by triple transfection of HEK293T/17 cells. An expression plasmid encoding the murine *Dnm2* cDNA (transcript variant 3, NCBI RefSeq NM_007871.2; lacking exons 12b and 13ter) was placed under the transcriptional control of the rat MPZ promoter. The constructs pAAV-rMPZ-mDNM2 and pAAV-CMV-MCS were co-transfected alongside the pHelper plasmid (Agilent, CA, USA) and the pAAV2/9 capsid plasmid (P0008, Penn Vector Core, PA, USA). Cells were harvested 48 h after transfection and lysed in the presence of Benzonase (100 U/mL, Merck), and viral particles were purified by iodixanol density gradient ultracentrifugation (OptiPrep™, Axis Shield). Purified fractions were subsequently dialyzed and concentrated in Dulbecco's PBS supplemented with 0.5 mM MgCl_2_ using Amicon Ultra-15 centrifugal devices (100 kDa molecular weight cut-off, Merck Millipore). Vector titres were determined by qPCR on a LightCycler 480 system using SYBR Green I Master mix (Roche), with primers directed against either the mDNM2 transgene or the CMV enhancer sequence (Supplementary Table 1). Final preparations were aliquoted and stored at −80°C.

### *In vivo* AAV injections

Intrathecal injections were carried out at 4 weeks of age under 1.5–2% isoflurane anaesthesia. After shaving and disinfecting the lumbar region, 10 µL of AAV9 was injected into the subarachnoid space between lumbar vertebrae L5 and L6 using a 27G needle connected to a Hamilton syringe, to a dose of 3.1 × 10^11^ gc per mouse. Correct needle placement was confirmed by the tail-flick reflex. Untreated control animals received equivalent volumes of empty AAV vector using the same injection procedure.

### Behavioural tests

Behavioural assessments were conducted at 7 weeks of age, on the same weekday and in the same order for all animals, by a single experimenter blinded to genotype and treatment.

Motor performance was assessed using a hanging (inverted grid) test, with latency to fall recorded up to 60 s. Each animal completed three trials, and the mean of the two best results was used for analysis. Gait parameters were captured on a motorized treadmill fitted with an underfloor camera, operating at a fixed speed of 10 cm/s. Both body stretch (nose-to-tail base distance) and stride length (step distance normalized to body stretch) were quantified; values represent the average of three measurements. Sensorimotor coordination was assessed with a notched bar test, in which animals traversed a horizontal bar featuring alternating notches. The number of falls and hindlimb slips into notches across 10 trials was recorded and averaged per mouse.

Mechanical sensitivity was measured using the Von Frey test. Calibrated filaments (0.6 g to 4 g) were applied perpendicularly to the plantar surface of the hind paw, recording rapid foot lifting as a positive reaction, and the 50% paw withdrawal threshold was determined using the up-down method^25^ and the up-down reader programme.^26^ Thermal sensitivity was measured using the hot plate (52°C), recording latency to hind paw withdrawal or licking. For tail immersion (48°C), latency to tail flick was measured. Mice were habituated to the restraint tube on day 1, and measurements were taken on day 2. Each test consisted of three trials with 5-min intervals, and values were averaged. Body length was measured post-mortem (nose to tail base).

### Electrophysiology

Mice were anaesthetized with isoflurane (induction at 3%, maintenance at 2%) in oxygen-enriched air. Body temperature was monitored via rectal probe and maintained between 34–37°C using a heating pad and a heat lamp. Eyes were protected with Ocrygel to prevent drying. EMG was performed using a Natus Mag2Health system. For sensitive evaluation, stimulating and recording electrodes were placed on the tail (separated by 2 cm), with the ground electrode in between. For motor nerve evaluation, needle electrodes were placed for proximal stimulation at the sciatic nerve and distal stimulation at the ankle. The ground electrode was inserted subcutaneously on the contralateral side, and recordings were obtained from the plantar muscle. Stimulation duration was 0.1 ms, and intensity was adjusted to elicit maximal responses.

### *In situ* muscle force

TA muscle contractile function was evaluated *in situ* at 8 weeks of age using the Aurora Scientific 1300A system. Anaesthesia was induced by intraperitoneal injection of domitor (2 mg/kg), fentanyl (0.28 mg/kg) and diazepam (8 mg/kg). Mice were placed under a heat source to maintain body temperature. The TA muscle was surgically exposed, and the distal tendon was attached to an isometric force transducer. The sciatic nerve was accessed through a small incision at the thigh, and stimulating electrodes were placed either around the nerve or directly on the muscle. Muscle length and stimulation intensity were optimized for maximal twitch force. Isometric tetanic force was first recorded following sciatic nerve stimulation (150 Hz, 0.5 s duration), then a force-frequency curve was generated by applying stimuli ranging from 1 to 150 Hz. Both measurements were repeated with direct muscle stimulation. A fatigue protocol was subsequently applied consisting of 80 consecutive trains (40 Hz, 1 s on/3 s off), and peak force was recorded for each contraction. For fatigue analysis, tetanic force was averaged every five contractions, and fatigue was expressed as the per cent decline in force from the first five to the last five contractions. Specific force (mN/mg) was obtained by normalizing force to TA muscle mass.

### Tissue collection

Muscles were dissected and weighed. TA muscles destined for histological or biochemical analyses were snap-frozen in liquid nitrogen-cooled isopentane and stored at −80°C. Sciatic nerves for molecular studies were snap-frozen in liquid nitrogen and kept at −80°C. For fibre bundle isolation (bungarotoxin immunofluorescence), the entire hindlimb (cut above the knee) was fixed with the leg extended in 4% PFA for 24 h at 4°C. The extensor digitorum longus (EDL) muscle was then dissected, rinsed in PBS (3x), incubated in 25% sucrose at 4°C until the tissue sank and stored at −80°C. Sciatic nerves for semi-thin sections were fixed in 2.5% glutaraldehyde and 2.5% paraformaldehyde in 0.1 M cacodylate buffer (pH 7.4) and stored at 4°C. For immunofluorescence, nerves were fixed flat on filter paper in 4% PFA for 24 h at 4°C, rinsed in PBS (3×), incubated in 25% sucrose at 4°C until the tissue sank and stored at −80°C.

### Muscle histology

Transversal cryostat sections of TA muscle (8 µm) were prepared and stained with haematoxylin and eosin (HE) by the IGBMC histology platform. Stained slides were scanned using a Carl Zeiss Axioscan 7, and 20× images were used for analysis. Fibre boundaries were delineated on HE-stained sections using CellPose^27^ software, and minimum Feret diameters were measured in Fiji.^28^ One transversal section of the whole muscle was quantified for each animal.

### Sciatic nerve histology

Fixed sciatic nerve samples were washed in cacodylate buffer (0.1 M, pH 7.4) for 30 min. Post-fixation was performed with 1% osmium tetroxide in 0.1 M cacodylate buffer for 1 h at 4°C. Samples were subsequently dehydrated through an ascending ethanol series (50%, 70%, 90%, 100%) and treated with propylene oxide, 30 min per step. Nerves were oriented transversely and embedded in Epon 812. Semithin sections (2 µm thick), performed as distally as possible, were stained with toluidine blue. Images were acquired at 63× magnification using a Zeiss Axio Observer 7 microscope. Axon categories and myelin abnormalities were classified across the entire nerve section using QuPath.^29^ g-ratio, axon diameter and myelin thickness were quantified on the whole stained section using the AxonDeepSeg segmentation software.^30^

### Muscle immunofluorescence

For bungarotoxin staining, EDL muscles were thawed on ice, and single fibres or small bundles were manually isolated under a stereomicroscope in cold PBS. Samples were mounted on slides, fixed in 4% PFA for 20 min, then permeabilized in PBS containing 0.5% Triton X-100 for 30 min at RT. Blocking was performed for 1 h in PBS with 1% BSA and 0.1% Triton X-100. Slides were incubated with bungarotoxin antibody and DAPI (Supplementary Table 1 Reagents) for 1.5 h at RT, then mounted with ProLong Gold Antifade. Images were acquired at 20× using a Zeiss Axio Observer 7 microscope. Neuromuscular junctions were detected on Fiji by adjusting the threshold, and signal area was measured.

### Sciatic nerve immunofluorescence

Longitudinal (10 µm) and transversal (8 µm) cryosections of sciatic nerve were fixed in 4% PFA for 20 min and permeabilized in 0.2% Triton X-100 in PBS (Merck #T8787-250ML) for 10 min. Non-specific binding was blocked for 1 h using 5% BSA (MP Bio #02160069-CF) in 0.1% Triton X-100/PBS. Primary antibodies (Supplementary Table 1 Reagents) were applied overnight at 4°C, followed by incubation with appropriate Alexa Fluor-conjugated secondary antibodies and DAPI for 1 h at room temperature. Sections were coverslipped using ProLong Gold Antifade reagent (ThermoFisher #P36934). Fluorescent images were obtained at 20× or 63× on a Zeiss Axio Observer 7 microscope. For each animal, sections processed without primary antibody served as negative controls. NF-H staining intensity was quantified in Fiji by measuring the mean grey value within the largest possible nerve area. The NF-H staining area represents the percentage of the nerve area covered by NF-H positive signal.

### Protein extraction

Muscle and nerve tissues were each lysed in dedicated RIPA formulations. Muscle lysis buffer contained 150 mM NaCl, 50 mM Tris (pH 8), 0.5% sodium deoxycholate, 1% NP-40, and 0.1% SDS; nerve lysis buffer comprised 2% SDS, 25 mM Tris (pH 8.0), 95 mM NaCl, 2 mM EDTA and 0.5% sodium deoxycholate. Both buffers were freshly supplemented with 1 mM PMSF, 1 mM sodium orthovanadate, 5 mM sodium fluoride and 1× protease inhibitor cocktail. Tissue homogenization was performed using a Precellys® Evolution Touch instrument (Bertin Technologies) in two 20 s cycles at 6000 rpm, followed by centrifugal clarification. Total protein concentration was quantified using the DC Protein Assay Kit (#5000116, BioRad).

### Western blotting

Proteins were separated on 10% polyacrylamide gels produced in-house. Loaded amounts were 10 µg (12 µL) for muscle samples and 5–12 µg (20 µL) for nerve samples. Electrophoresis was run at 130 V for approximately 1 h, and proteins were subsequently transferred to nitrocellulose membranes using the Trans-Blot Turbo RTA Mini Nitrocellulose kit (#170-4270, BioRad) at 2.5 A for 5–10 min. Membranes were stained with Ponceau S and blocked for 1 h in 5% non-fat dry milk dissolved in TBS containing 0.1% Tween-20 (TBST; #P2287-500ML, Merck). Primary antibodies (Supplementary Table 1 Reagents) were diluted in TBST/5% milk and applied overnight at 4°C. After washing, membranes were incubated for 1 h at RT with HRP-conjugated secondary antibodies. Immunoreactive bands were visualized by enhanced chemiluminescence (#32209, ThermoFisher Scientific) and recorded on an Amersham Imager 600 (GE Healthcare Life Sciences). Band densitometry was performed in Fiji; values were normalized to Ponceau S staining and expressed relative to the mean of the control group. All unprocessed, full-length western blot images used for quantification, including those not displayed in the main figures, along with their corresponding loading controls, are provided as Supplementary material.

### Statistical analysis

All statistical analyses and graph generation were carried out using GraphPad Prism software (version 10.0.2). In our study, males and females were initially analysed separately to assess potential sex effects. Two-way ANOVA revealed a significant effect of sex and a sex × genotype interaction only for body mass and the hanging test; these data are therefore presented by sex. For all other analyses, no significant sex effect or no sex × genotype interaction was detected, and data were pooled while ensuring balanced representation of both sexes across groups as much as possible (see Supplementary Table 2: Statistics). Data distribution was first assessed by the Shapiro–Wilk normality test. Normally distributed data with homogeneous variances were compared by one-way ANOVA with Tukey’s *post hoc* correction. When variances were unequal, a Brown–Forsythe and Welch ANOVA followed by Dunnett’s T3 *post hoc* test was applied. When data followed a log-normal distribution, a log transformation was performed prior to analysis. Non-normally distributed datasets were analysed using the Kruskal–Wallis test with Dunn’s *post hoc* test. Two-way ANOVA was used when two factors were assessed (e.g. body mass × sex; force × stimulation frequency; axon distribution × diameter category; body mass or hanging time × age) followed by Tukey’s or Bonferroni’s multiple-comparison tests. All pairwise comparisons were computed, but only statistically significant differences are indicated on the graphs. Statistical significance was defined as *P* < 0.05. Graphs display individual data points with mean ± SD. Additional details on statistical tests and sample sizes are given in Supplementary Table 2.

## Results

### Severe and irreversible motor defects in the *DNM2*–CMT mouse model

To evaluate the therapeutic potential of nerve-targeted DNM2 supplementation, we used the severe *Dnm2^K562E/^*^SC−^ mouse model. These mice carry systemic heterozygosity for the K562E mutation and lack WT *Dnm2* specifically in Schwann cells. Previous studies showed that expression of the K562E allele alone is sufficient to support correct radial sorting and initiation of myelination at postnatal day 5. However, it is not sufficient to maintain the myelinated state of Schwann cells, as by postnatal day 24 these mice display a marked reduction in myelinated axons and severe electrophysiological deficits.^21^ Notably, only ultrastructural and electromyography (EMG) analyses were reported in these mice; we thus performed here a comprehensive phenotypic characterization.

As AAV9-mediated gene therapies have proven effective in both demyelinating and axonal forms of CMT,^31,32^ and intrathecal AAV9 delivery under the Schwann cell-specific MPZ promoter efficiently targets the peripheral nervous system,^33^ we delivered murine DNM2 under the control of the rat MPZ promoter intrathecally using AAV9 at a dose of 3.1 × 10^11^ genome copies per mouse, administered at 4 weeks of age after symptom onset (Supplementary Fig. 1). Behavioural analyses were performed at 7 weeks, followed by functional tests and tissue sampling at 8 weeks (Supplementary Fig. 2).

First, we observed by western blot only a slight decrease in the mean DNM2 levels in the sciatic nerve of untreated *Dnm2^K562E/^*^SC−^ compared to controls (0.74-fold), likely due to the presence of other cell types (e.g. fibroblasts, endothelial cells, immune cells) (Supplementary Fig. 3A, 10). Then, we validated a significant increase in DNM2 levels in the sciatic nerve of treated *Dnm2^K562E/^*^SC−^ mice (1.4-fold versus controls, 1.9-fold versus untreated *Dnm2^K562E/^*^SC−^). Unexpectedly, treated control WT mice showed a decrease in DNM2 expression, which does not preclude investigating the therapeutic potential of DNM2 overexpression in the *DNM2*–CMT model.

**Figure 1:**
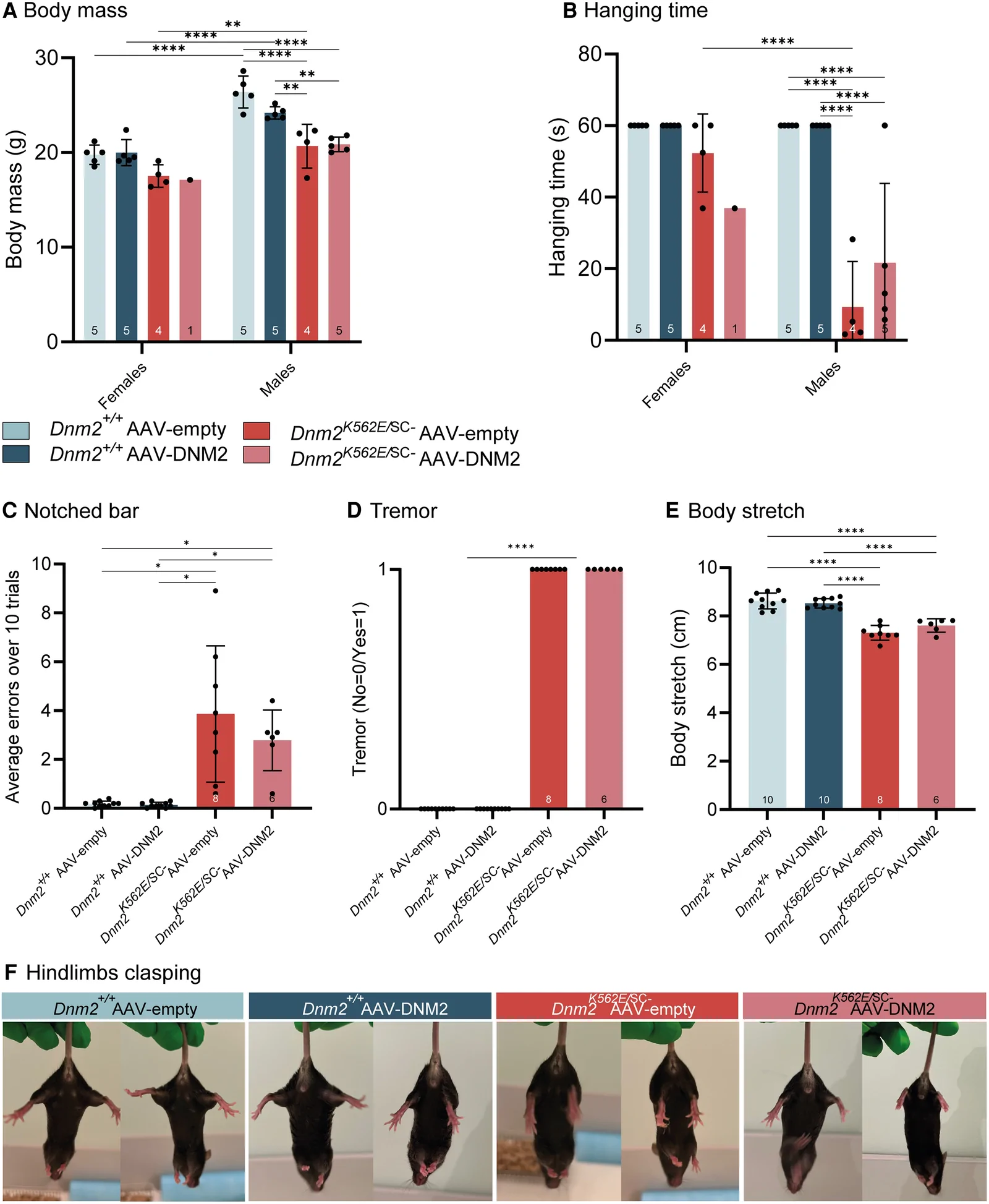
**Severe and irreversible motor defects in the *DNM2*-CMT mouse model.** (**A**) Body mass of females and males at 8 weeks. Two-way ANOVA (interaction *F* = 2.846 *P* = 0.057, sex *F* = 69.13, *P* < 0.0001, genotype *F* = 17.27 *P* < 0.0001) with Tukey’s multiple comparisons. (**B**) Hanging test performance of females and males at 7 weeks. Maximum hanging time = 60 s. Two-way ANOVA (interaction *F* = 8.193 *P* = 0.0005, sex *F* = 12.58, *P* = 0.0015, genotype *F* = 19.66 *P* < 0.0001) with Tukey’s multiple comparisons. (**A–B**) N females: *Dnm2*^+/+^-empty, *n* = 5; *Dnm2*^+/+^-DNM2, *n* = 5; *Dnm2^K562E/^*^SC−^-empty, *n* = 4; *Dnm2^K562E/^*^SC−^-DNM2, *n* = 1. *N* males: *Dnm2*^+/+^-empty, *n* = 5; *Dnm2*^+/+^-DNM2, *n* = 5; *Dnm2^K562E/^*^SC−^-empty, *n* = 4; *Dnm2^K562E/^*^SC−^-DNM2, *n* = 5. (**C)** Average number of errors (hindlimb slips) when crossing the notched bar over 10 trials at 7 weeks. Brown-Forsythe ANOVA test (F* = 12.50, *P* = 0.0011) with Dunnett’s T3 multiple comparisons. (**D**) Presence (1) or absence (0) of tremor at 7 weeks. Chi-square test (Chi-square = 34.00, df = 3, *P* < 0.0001). (**E**) Body stretch (nose to tail base length) measured during treadmill walking at 7 weeks. Ordinary one-way ANOVA (*F* = 47.24, *P* < 0.0001) with Tukey’s multiple comparisons. (C-E) N: *Dnm2*^+/+^-empty, *n* = 10; *Dnm2*^+/+^-DNM2, *n* = 10; *Dnm2^K562E/^*^SC−^-empty, *n* = 8; *Dnm2^K562E/^*^SC−^-DNM2, *n* = 6. (**F**) Tail suspension at 7 weeks, indicating presence or absence of hindlimb clasping (*n* = 2 mice per group). Each dot represents a mouse. Charts present individual values and mean ± standard deviation, *P < 0.05, **P < 0.01, ****P < 0.0001.

*Dnm2^K562E/^*^SC−^ mice exhibited reduced body size at 7 weeks (Supplementary Fig. 3B) and reduced body mass especially in males (Fig. 1A; Supplementary Fig. 1C). They also showed impaired motor performance, with a 6-fold decrease in hanging time in males compared to controls and increased coordination deficits in both sexes, reflected by a higher number of errors and falls on the notched bar test (Fig. 1B–C; Supplementary Figs 1D and 3C). All *Dnm2^K562E/^*^SC−^ mice displayed tremor (Video 1, Fig. 1D). Despite these deficits, the *Dnm2^K562E/^*^SC−^ mice remained notably active in their cages and did not display signs of paralysis (Video 2). *Dnm2^K562E/^*^SC−^ mice showed a 1.3-cm reduction in body stretch during walking, consistent with shorter body length and increased stride length, further supporting coordination defects (Fig. 1E; Supplementary Fig. 3B and D). Hindlimb clasping during tail suspension was also observed, suggestive of underlying neurodegeneration (Fig. 1F). Thermal and mechanical sensitivity, assessed by hot plate, tail immersion and Von Frey tests at 7 weeks, revealed no deficits in *Dnm2^K562E/^*^SC−^ mice (Supplementary Fig. 3E–G).

Three weeks of DNM2 overexpression in peripheral nerves, initiated at 4 weeks of age, did not reverse the severe motor and coordination deficits in *Dnm2^K562E/^*^SC−^ mice, supporting that under these conditions of treatment these deficits are not reversible.

### The *Dnm2^K562E/^*^SC−^ model exhibits severe CMT-like nerve conduction impairments

To further investigate the neuromuscular dysfunction in this *DNM2*–CMT mouse model and evaluate potential rescue at the functional level following DNM2 supplementation, we next performed EMG and *in situ* muscle force measurements at 8 weeks.

**Figure 2:**
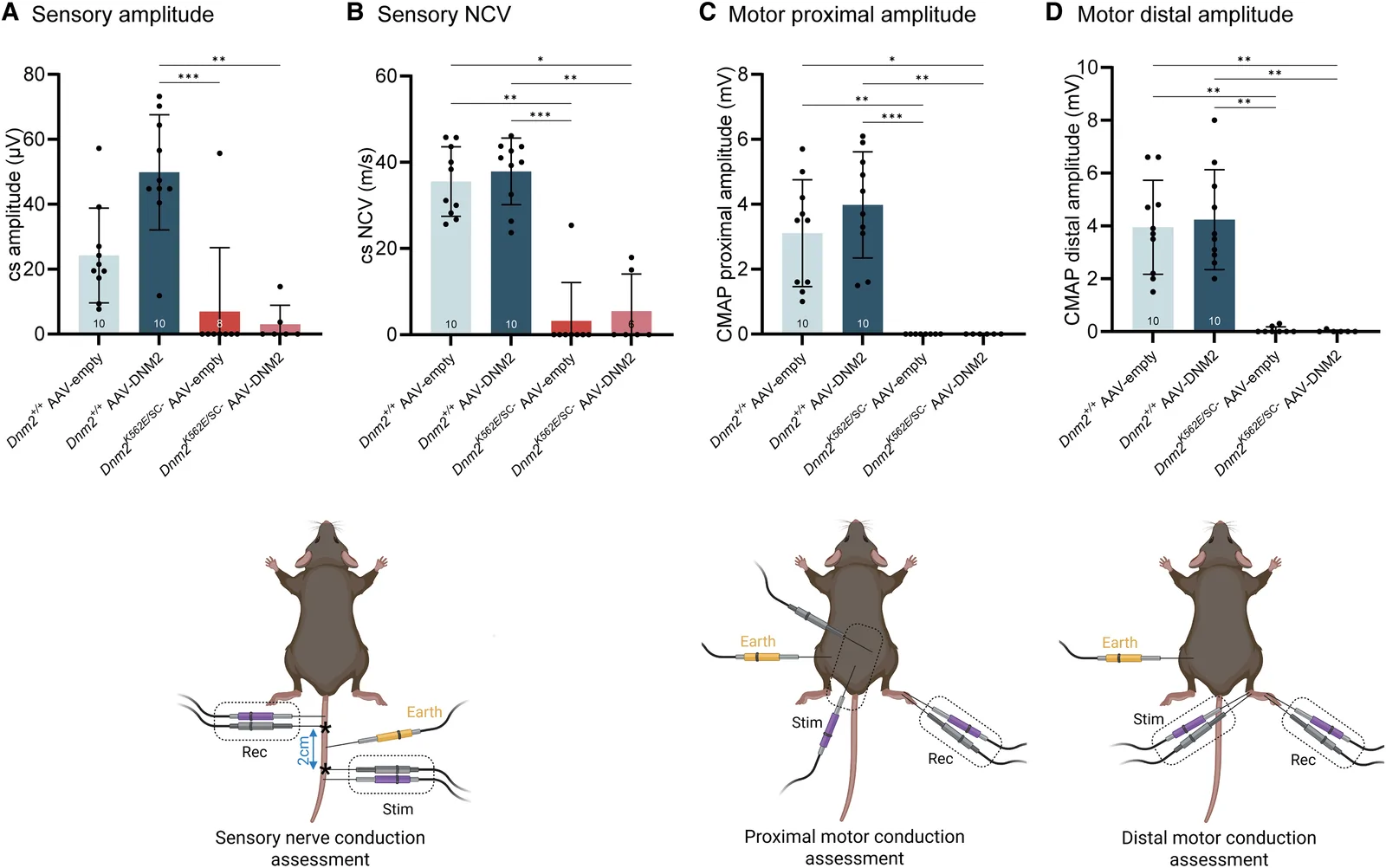
**The *Dnm2^K562E/^*^SC*−*^ model exhibits severe CMT-like nerve conduction impairments.** (A-B) Sensory electrophysiology at 8 weeks: (**A**) compound sensory (cs) amplitude and, (**B**) nerve conduction velocity (NCV). (C-D) Motor electrophysiology at 8 weeks: (**C**) proximal compound muscle action potential (CMAP) and, (**D**) distal CMAP. Stim = stimulation, Rec = recording electrodes. Created using BioRender. Laporte, J. (2026). https://BioRender.com/tt8jcdf. (A-D) Kruskal-Wallis (*P* < 0.0001) with Dunn’s multiple comparisons. . (A-D) *N*: *Dnm2^+/+^*-empty, *n* = 10; *Dnm2^+/+^*-DNM2, *n* = 10; *Dnm2^K562E/^*^SC−^-empty, *n* = 8; *Dnm2^K562E/^*^SC−^-DNM2, *n* = 6. Each dot represents a mouse. Charts present individual values and mean ± standard deviation, *P < 0.05, **P < 0.01, ***P < 0.001.

EMG revealed markedly reduced or absent sensory nerve conduction amplitude and velocity in *Dnm2^K562E/^*^SC−^ mice (Fig. 2A–B). Proximal and distal motor responses were undetectable, preventing calculation of motor nerve conduction velocity (Fig. 2C–D). DNM2 increase in peripheral nerves through AAV-DNM2 intrathecal injection did not improve any of these electrophysiological parameters. This pronounced reduction in sensory nerve conduction velocity did not translate into detectable deficits in sensory assays (Supplementary Fig. 3E–G), which may reflect differences in the fibre populations assessed: electrophysiological recordings predominantly evaluate large myelinated fibres, whereas thermal nociceptive tests mainly stimulate unmyelinated C fibres.

**Figure 3:**
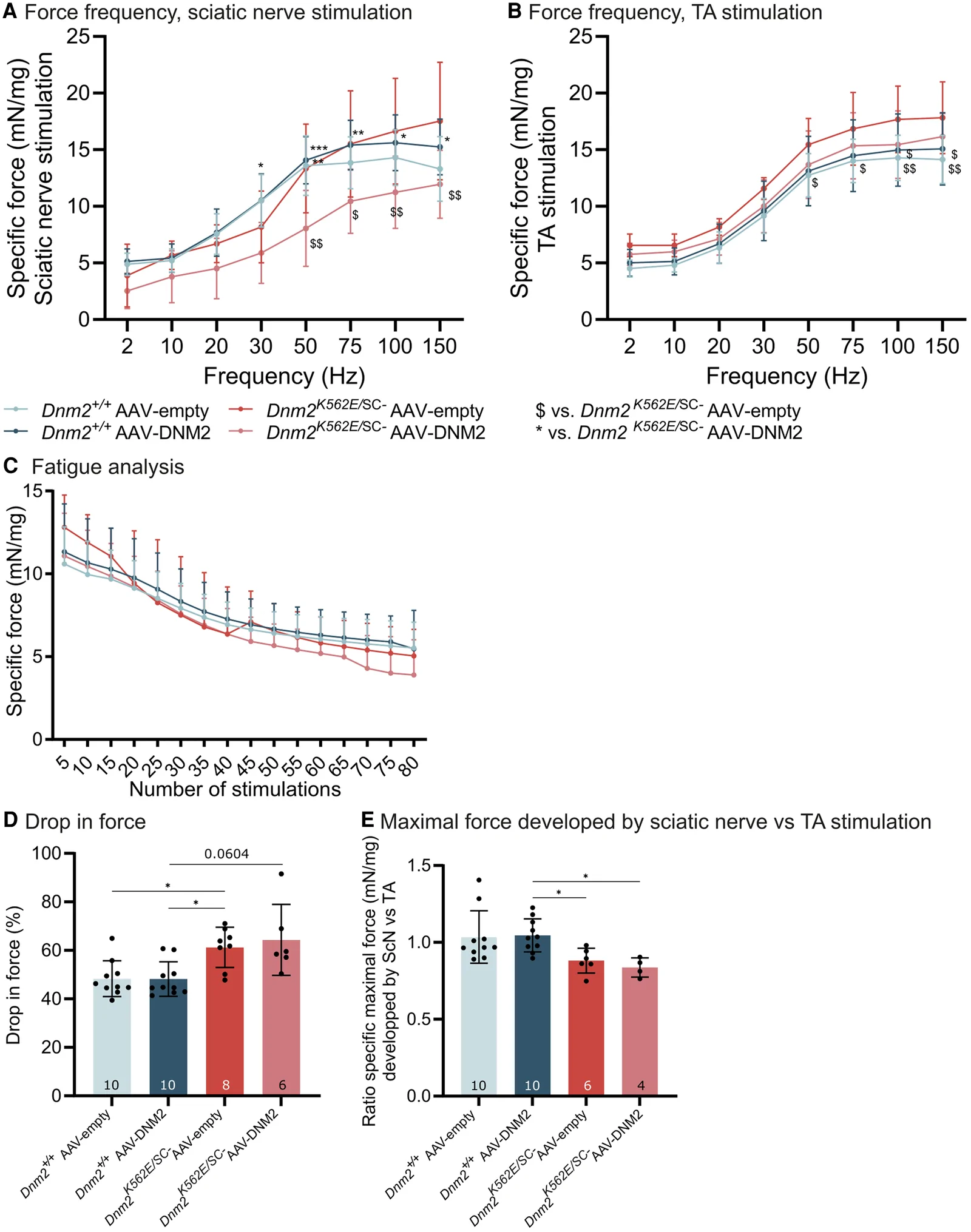
**The *Dnm2^K562E/^*^SC*−*^ model shows no intrinsic muscle weakness but nerve conduction-dependent deficits.** (**A**) Force developed by Tibialis Anterior (TA) muscle when stimulating the sciatic nerve. Two-way ANOVA (interaction *F* = 1.152, *P* = 0.2971, Frequency *F* = 75.15, *P* < 0.0001, genotype *F* = 18.73, *P* < 0.0001) with Tukey’s multiple comparisons. (**B**) Force developed by TA muscle when stimulating the TA at different incremental frequencies (1–150 Hz) at 8 weeks, normalized to TA mass. Two-way ANOVA (interaction *F* = 0.2002, *P* > 0.9999, Frequency *F* = 142.7, *P* < 0.0001, genotype *F* = 18.46, *P* < 0.0001) with Tukey’s multiple comparisons. In (**A–B**), significant differences between *Dnm2^K562E/^*^SC−^ AAV-empty and other groups were indicated with $, while * shows differences between *Dnm2^K562E/^*^SC−^ AAV-DNM2 and other groups. (**C**) Force developed by TA muscle over 80 consecutive TA stimulations at 40 Hz, normalized to TA mass. Two-way ANOVA (interaction *F* = 0.4562, *P* = 0.9991, Stimulations *F* = 38.20, *P* < 0.0001, genotype *F* = 5.017, *P* = 0.0020) with Tukey’s multiple comparisons. (**D**) Drop in force between five first and five last stimulations during fatigue analysis at 40 Hz. Kruskal–Wallis (*P* = 0.0025) with Dunn’s multiple comparisons. (**E**) Ratio of the maximal force developed from sciatic nerve versus TA muscle stimulation. Kruskal–Wallis (*P* = 0.0035) with Dunn’s multiple comparisons. (**B–D**) *N*: *Dnm2^+/+^*-empty, *n* = 10; *Dnm2^+/+^*-DNM2, *n* = 10; *Dnm2^K562E/^*^SC−^-empty, *n* = 8; *Dnm2^K562E/^*^SC−^-DNM2, *n* = 6. (**A**, **E**) N: *Dnm2^+/+^*-empty, *n* = 10; *Dnm2^+/+^*-DNM2, *n* = 10; *Dnm2^K562E/^*^SC−^-empty, *n* = 6; *Dnm2^K562E/^*^SC−^-DNM2, *n* = 4. Each dot represents a mouse. Charts present individual values and mean ± standard deviation, *P < 0.05, **P < 0.01, ***P < 0.001.

*In situ* force measurements revealed no relevant difference in Tibialis Anterior (TA) muscle force upon sciatic nerve stimulation among the groups (Fig. 3A). When stimulating the muscle directly, force output appeared slightly higher in mutants compared to controls, but this was likely due to normalization to a reduced muscle mass caused by atrophy (Fig. 3B). Fatigue exercise showed a greater decline in force production throughout the test in untreated mutants compared to controls (Fig. 3C–D). Although no intrinsic muscle weakness was evident, force generated through sciatic nerve stimulation tended to be lower than via direct muscle stimulation in *Dnm2^K562E/^*^SC−^ mice (Fig. 3E), suggesting conduction impairments.

Taken together, functional assessments showed no intrinsic muscle weakness but clear nerve conduction impairments in *Dnm2^K562E/^*^SC−^ mice, with no improvement following DNM2 intrathecal delivery. The *Dnm2^K562E/^*^SC−^ mice thus reproduce a severe peripheral neuropathy alike in *DNM2*–CMT patients.

### The *Dnm2^K562E/^*^SC−^ model reproduces myelination deficits and axonal pathology characteristic of *DNM2*–CMT

**Figure 4:**
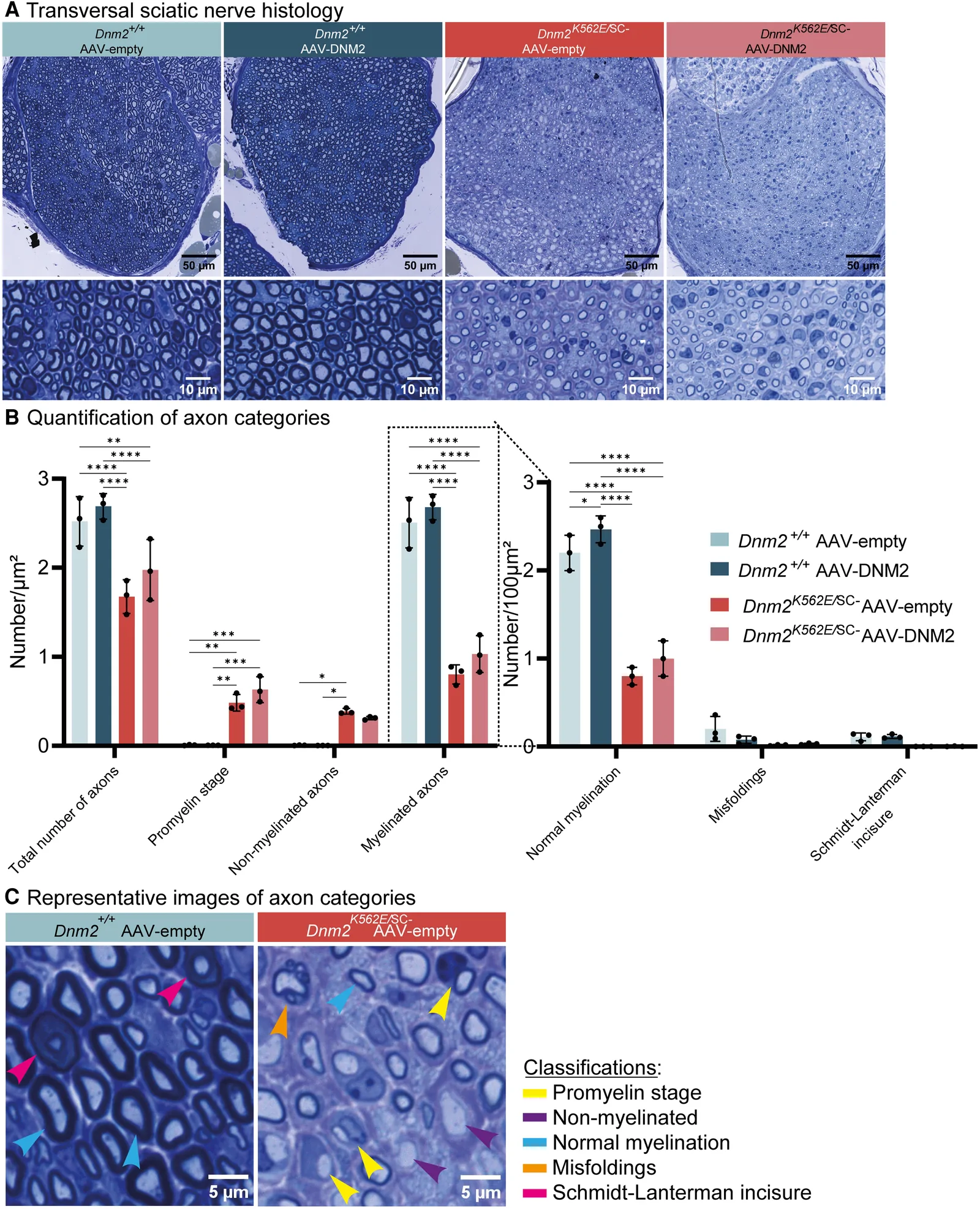
**The *Dnm2^K562E/^*^SC*−*^ model reproduces myelination deficits and axonal pathology characteristic of *DNM2*-CMT.** (**A**) Transversal section of the entire sciatic nerve stained with toluidine blue (upper panel, scale bar = 50 µm); magnified view in lower panel (scale bar = 20 µm). (**B**) Quantification of axon categories in transversal sciatic nerve sections per 100µm^2^ at 8 weeks. *n* = 3 mice per group. Two-way ANOVA (left graph: interaction *F* = 40.26, *P* < 0.0001, axon-type *F* = 468.8, *P* < 0.0001, genotype *F* = 25.54, *P* < 0.0001; right graph: interaction *F* = 52.86, *P* < 0.0001, axon-type *F* = 834.9, *P* < 0.0001, genotype *F* = 81.15, *P* < 0.0001) with Tukey’s multiple comparisons. (**C**) Representative images illustrating the classification of axon categories used for quantification in (**B**). Scale bar = 5 µm. Each dot represents a mouse. Charts present individual values and mean ± standard deviation, *P < 0.05, **P < 0.01, ***P < 0.001, ****P < 0.0001.

To characterize the cellular basis for the nerve conduction impairments in the severe *DNM2*–CMT model, we investigated the structure and molecular biomarkers of the peripheral nerves. Semi-thin transversal sections of sciatic nerves stained with toluidine blue qualitatively revealed increased endoneurial connective tissue, signs of demyelination and reduced fibre size (Fig. 4A). Quantifications showed a decreased number of total axons in the *Dnm2^K562E/^*^SC−^ mice with no improvement upon DNM2 overexpression (Fig. 4B). Mutant sections exhibit an increased number of fibres in a promyelinating stage, defined by Schwann cell nuclei positioned directly adjacent to axons (yellow arrow), a higher number of non-myelinated axons (purple arrow) almost absent in control groups and a decrease number of myelinated axons (blue arrow) (Fig. 4C). Among myelinated axons, their overall number was reduced in mutants, but the frequency of structural myelin abnormalities (orange and pink arrows) was normal. DNM2 supplementation did not improve these histological defects.

**Figure 5:**
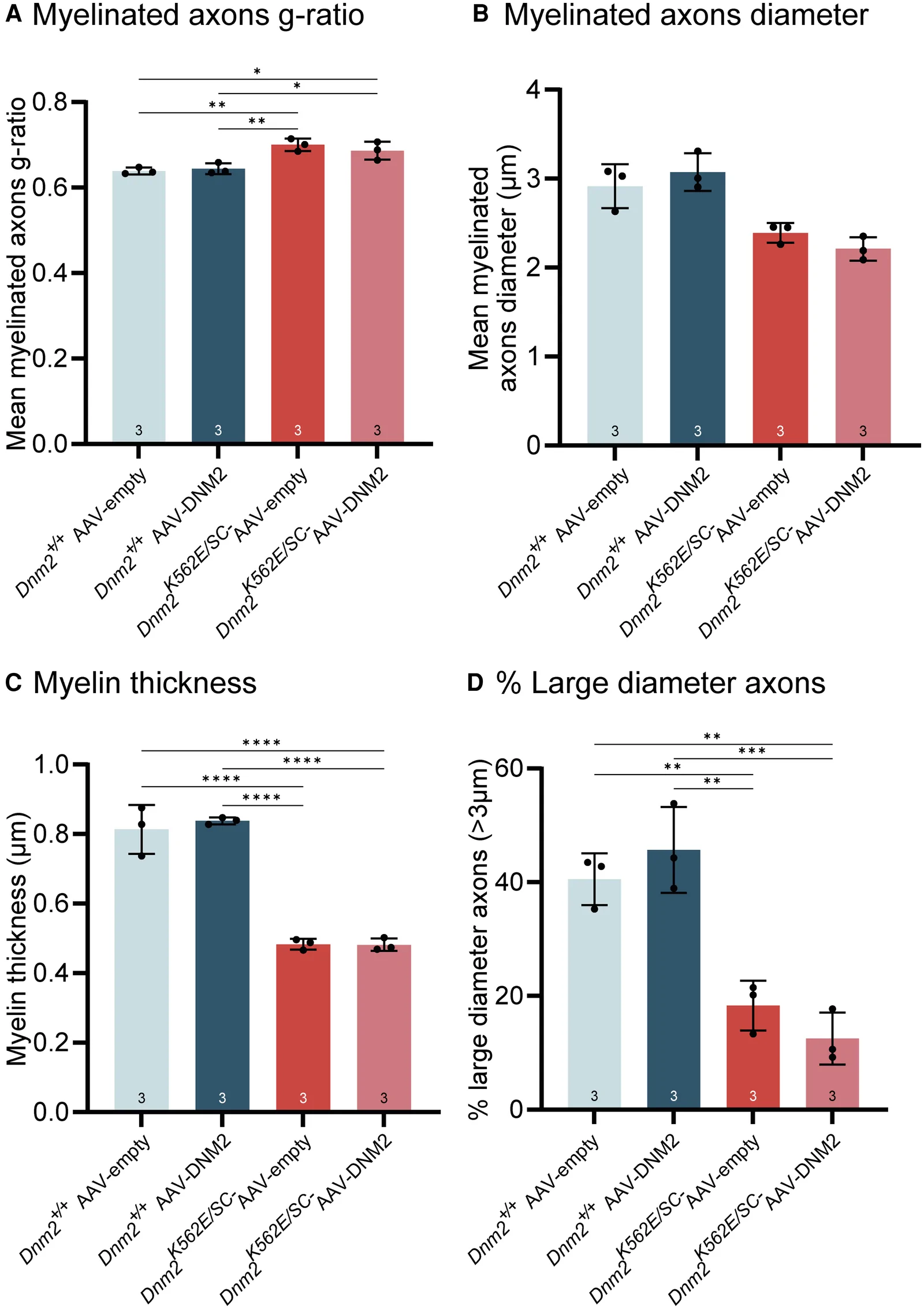
**The *Dnm2^K562E/^*^SC*−*^ model exhibits myelination defects and loss of large myelinated axons that are refractory to DNM2 supplementation.** (**A**) Mean g-ratio $\left(=\frac{axon\;diameter}{axon+myelin\;diameter}\right)$ of myelinated sciatic nerve fibres at 8 weeks. *n* = 3 mice per group. Ordinary one-way ANOVA (*F* = 12.56, *P* = 0.0021) with Tukey’s multiple comparisons. (**B**) Mean axon diameter of myelinated sciatic nerve fibres at 8 weeks. *n* = 3 mice per group. Kruskal–Wallis (*P* = 0.0039) with Dunn’s multiple comparisons. (**C**) Mean myelin thickness of myelinated sciatic nerve fibres at 8 weeks. *n* = 3 mice per group. Ordinary one-way ANOVA (*F* = 84.04, *P* < 0.0001) with Tukey’s multiple comparisons. (**D**) Percentage of large axons (>3 µm) in sciatic nerve sections. *n* = 3 mice per group. Ordinary one-way ANOVA (*F* = 27.11, = 0.0002) with Tukey’s multiple comparisons. Each dot represents a mouse. Charts present individual values and mean ± standard deviation, *P < 0.05, **P < 0.01, ***P < 0.001, ****P < 0.0001.

Among myelinated axons, the mean g-ratio $\left(=\;\frac{axon\;diameter}{axon+myelin\;diameter}\right)$ was increased in mutants, reflecting predominantly thinner myelin rather than reduced axon size, and this was not improved following DNM2 delivery (Fig. 5A–C). Scatter plots of g-ratio versus axon diameter showed consistently higher g-ratios in *Dnm2^K562E/^*^SC−^ mice across axon sizes compared to controls (Supplementary Fig. 4A), indicating relative myelin thinning. In controls, myelin thickness increased with axon diameter (∼17% per 1 μm), while in mutants this relationship was lost (slope near zero), suggesting disrupted coordination between axon size and myelin thickness (Supplementary Fig. 4B). In addition, the scatter plots revealed markedly fewer axons above ∼3–4 µm in *Dnm2^K562E/^*^SC−^ mice. Quantification confirmed a loss of large-calibre myelinated axons (Fig. 5D), similar to what is observed in patients. These parameters were unchanged following DNM2 supplementation.

Overall, the severe nerve conduction impairments of the *Dnm2^K562E/^*^SC−^ mouse is due to a decrease in myelination and axon loss.

### Molecular profiling reveals defects in myelination and inflammation that are refractory to DNM2 supplementation

**Figure 6:**
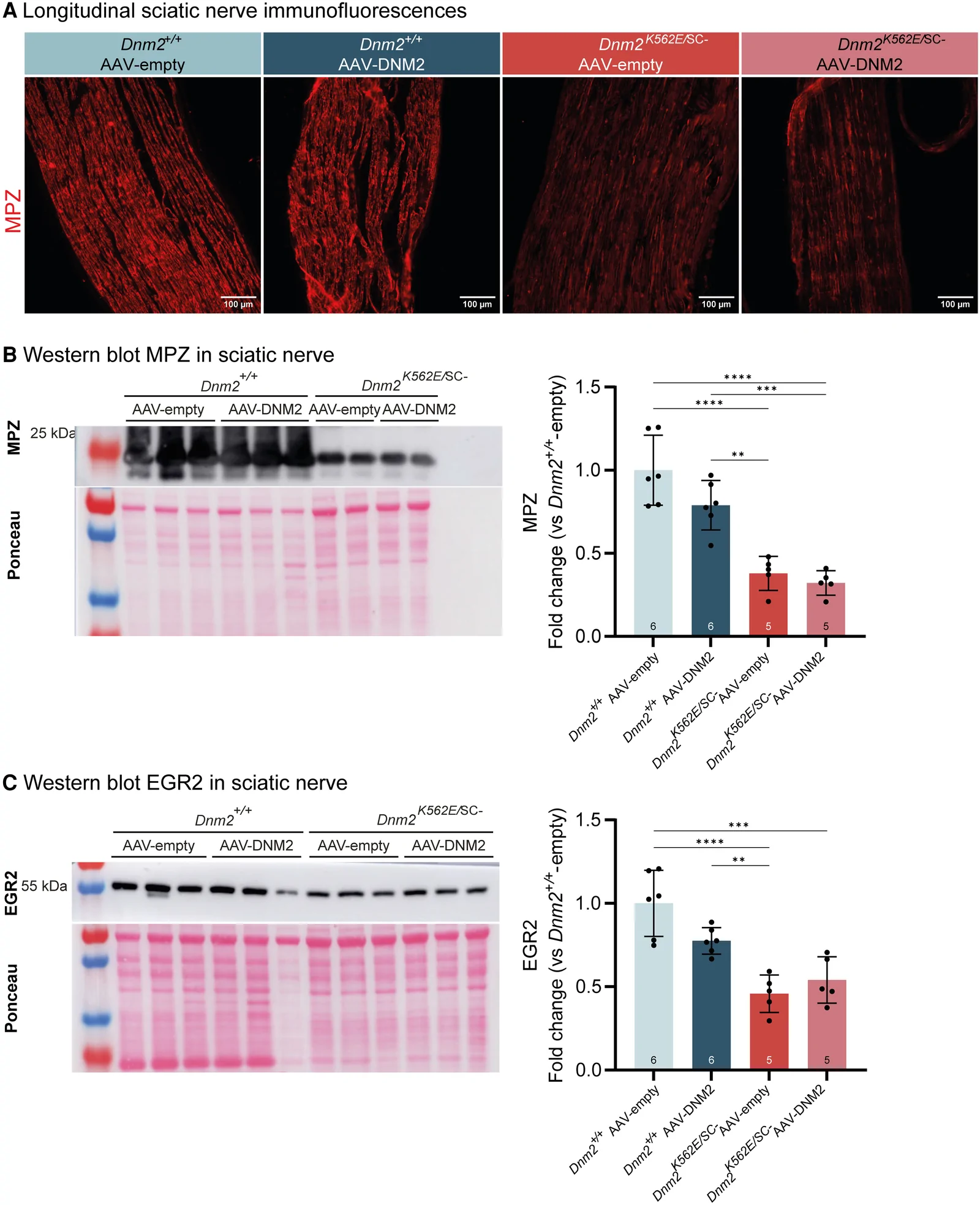
**The *Dnm2^K562E/^*^SC*−*^ model exhibits persistent molecular defects in myelination despite DNM2 supplementation.** (**A**) Longitudinal sections of sciatic nerve immunolabelled for Myelin Protein Zero (MPZ). Scale bar = 100 µm. (**B**) Representative western blot and quantification of MPZ proteins level in sciatic nerves at 8 weeks, normalized to Ponceau S staining. Ordinary one-way ANOVA (*F* = 26.49, *P* < 0.0001) with Tukey’s multiple comparisons. Uncropped images of the blots are shown in Supplementary material. (**C**) Representative western blot and quantification of EGR2 proteins level in sciatic nerves at 8 weeks, normalized to Ponceau S staining. Ordinary one-way ANOVA (*F* = 16.73, *P* < 0.0001) with Tukey’s multiple comparisons. Uncropped images of the blots are shown in Supplementary material. (**B–C**) *N*: *Dnm2^+/+^*-empty, *n* = 6; *Dnm2^+/+^*-DNM2, *n* = 6; *Dnm2^K562E/^*^SC−^-empty, *n* = 5; *Dnm2^K562E/^*^SC−^-DNM2, *n* = 5. Each dot represents a mouse. Charts present individual values and mean ± standard deviation, **P < 0.01, ***P < 0.001, ****P < 0.0001.

To identify the molecular alterations underlying these structural defects, we performed several molecular analyses. Immunofluorescence on longitudinal sciatic nerve sections revealed reduced MPZ signal intensity in *Dnm2^K562E/^*^SC−^ mice compared to controls (Fig. 6A). MPZ is the most abundant structural protein of peripheral myelin and is essential for compact myelin formation and maintenance. Its reduction was confirmed by Western blot, which showed a 62% decrease in MPZ protein levels compared to controls (Fig. 6B, Supplementary Fig. 8A), consistent with the myelin thinning observed (Fig. 5C) and impaired neural transmission (Fig. 2A–D). We also detected a 54% reduction in the transcription factor EGR2 compared to controls (Fig. 6C, Supplementary Fig. 8B). Given its role in activating myelin genes such as MPZ^34^ and in promoting the transition of promyelinating Schwann cells into fully myelinating cells,^35^ its downregulation likely contributes to the demyelination and abnormal Schwann cell nuclear positioning observed. Neither MPZ nor EGR2 levels were restored by DNM2 overexpression.

**Figure 7:**
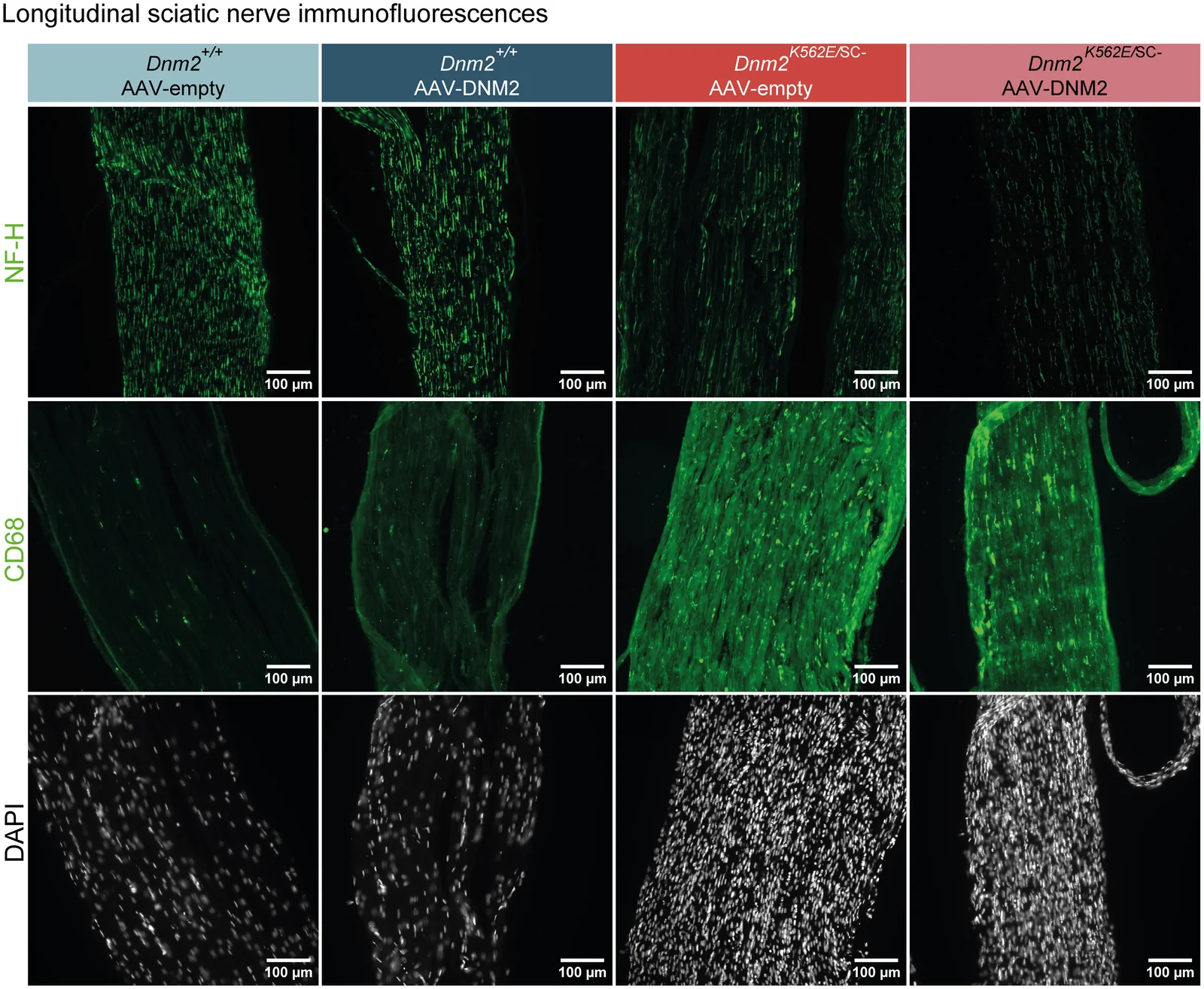
**Inflammatory and axonal features in sciatic nerves of *Dnm2^K562E/^*^SC*−*^ mice.** Longitudinal sections of sciatic nerves immunolabelled for Neurofilament Heavy (NF-H) (upper panel), CD68 (second panel) and DAPI (lower panel). *Dnm2^K562E/^*^SC−^ nerves exhibited reduced NF-H staining and increased CD68 signal and nuclei density compared with controls. Scale bar = 100 µm.

**Figure 8:**
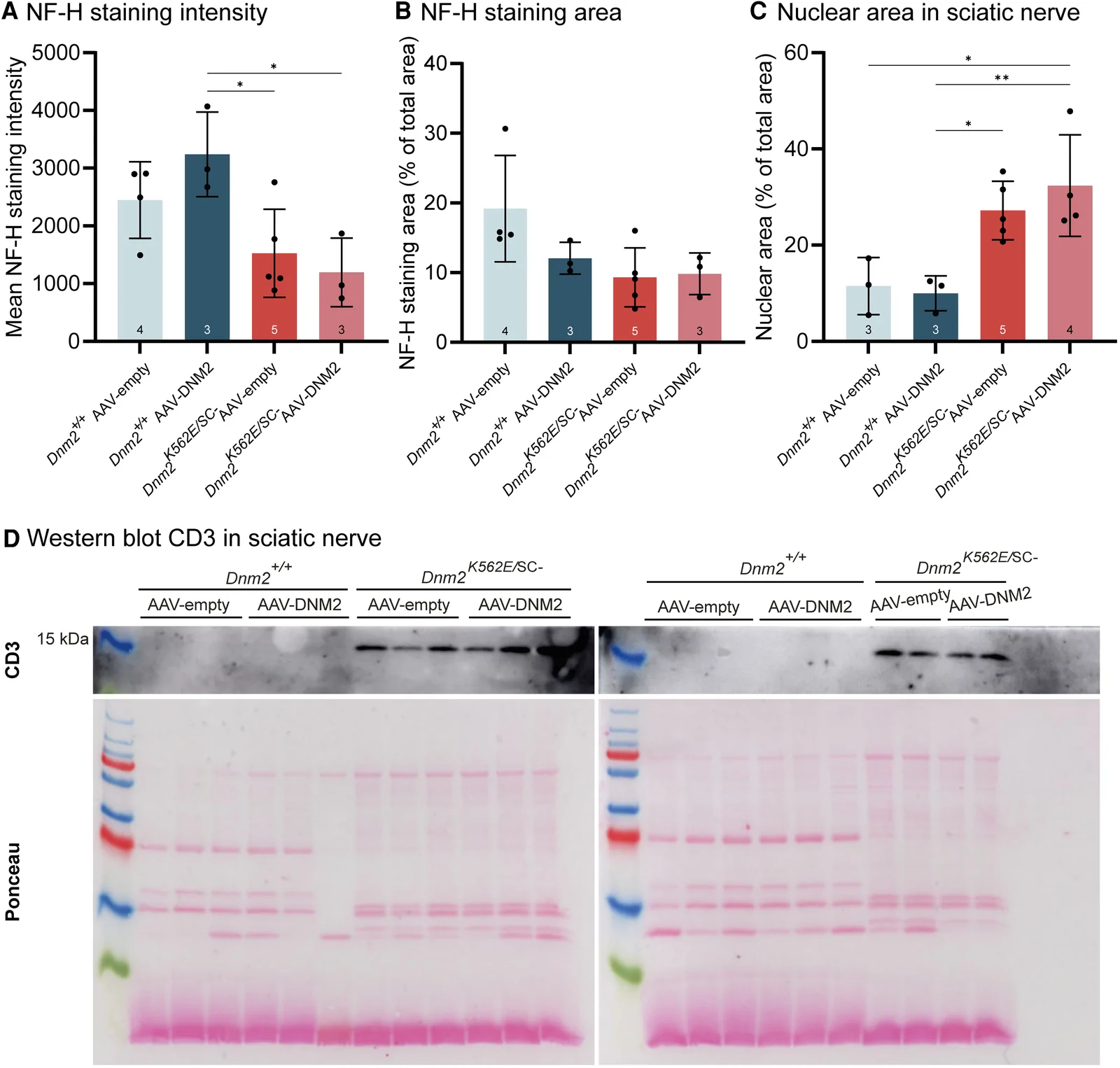
**Schwann cell-specific DNM2 supplementation is insufficient to correct inflammatory features in a severe model of *DNM2-*CMT.** (**A–B**) Quantification of neurofilament-heavy (NF-H) staining (**A**) intensity (Ordinary one-way ANOVA (*F* = 5.702, *P* = 0.0132) with Tukey’s multiple comparisons) and (**B**) area (Kruskal–Wallis (*P* = 0.0618) with Dunn’s multiple comparisons) in longitudinal sciatic nerve sections. *N*: *Dnm2^+/+^*-empty, *n* = 4; *Dnm2^+/+^*-DNM2, *n* = 3; *Dnm2^K562E/^*^SC−^-empty, *n* = 5; *Dnm2^K562E/^*^SC−^-DNM2, *n* = 3. (**C**) Nuclear area in longitudinal sciatic nerve sections, calculated as DAPI-positive area relative to total nerve area. *Dnm2^+/+^*-empty, *n* = 3; *Dnm2^+/+^*-DNM2, *n* = 3; *Dnm2^K562E/^*^SC−^-empty, *n* = 5; *Dnm2^K562E/^*^SC−^-DNM2, *n* = 4. Ordinary one-way ANOVA (*F* = 8.432, *P* = 0.0034) with Tukey’s multiple comparisons. (**D**) Representative western blot of CD3 protein in sciatic nerves at 8 weeks. Ponceau S staining is shown as a loading control. Uncropped images of the blots are shown in Supplementary material. Each dot represents a mouse. Charts present individual values and mean ± standard deviation, *P < 0.05, **P < 0.01.

We also performed immunofluorescence for NF-H (neurofilament heavy chain), a cytoskeletal component of large-calibre axons and a marker of axonal integrity (Fig. 7, upper panel). Both staining intensity and NF-H-positive area tended to be reduced in treated and untreated *Dnm2^K562E/^*^SC−^ nerves compared to controls (Fig. 8A–B), consistent with the overall axon loss observed in transversal sections (Fig. 4B).

As SC-specific loss of *Dnm2* is associated with inflammatory features,^18^ and because tissue injury typically triggers recruitment of immune cells, we performed immunofluorescence for CD68, a marker of macrophages mediating phagocytic clearance.^36^ CD68 staining revealed brighter signal intensity in *Dnm2^K562E/^*^SC−^ compared to controls (Fig. 7, second panel). This was accompanied by a notable increase in nuclei density, indicative of immune cell infiltration (Fig. 7, lower panel; Fig. 8C). To further characterize immune involvement, we assessed CD3 expression, a marker of T lymphocytes, in sciatic nerve lysates by western blot (Fig. 8D, Supplementary Fig. 9A). CD3 was observed in mutant nerves, both treated and untreated, whereas no CD3 signal was detected in control nerves, supporting lymphocytic infiltration in mutant nerves. Notably, intrathecal DNM2 delivery did not reduce these inflammatory changes. While immune cells play vital roles in tissue repair and regeneration,^36^ suppression of macrophages or depletion of mature T lymphocytes has been shown to lessen disease severity in related CMT models, suggesting that chronic or dysregulated inflammation can contribute to neuropathology.^37-41^

To ensure that these observations were not dependent on the anatomical region of the sciatic nerve, we analysed immunofluorescence at distinct portions of the longitudinal nerve sections, confirming that reductions in MPZ and NF-H staining were consistent across regions within the same animal (Supplementary Fig. 5A). In addition, transversal mid-nerve sections were taken at a standardized mid-nerve location in all groups and further confirmed our findings (Supplementary Fig. 5B). Similar results were observed for CD68 and nuclei infiltration (Supplementary Fig. 6).

Together, these molecular findings highlight a combined defect in myelin, axonal maintenance and inflammatory homeostasis in the peripheral nerves of *Dnm2^K562E/^*^SC−^ mice that were not rescued by DNM2 overexpression.

### The *Dnm2^K562E/^*^SC−^ model displays muscle atrophy that persists despite nerve-targeted DNM2 supplementation

**Figure 9:**
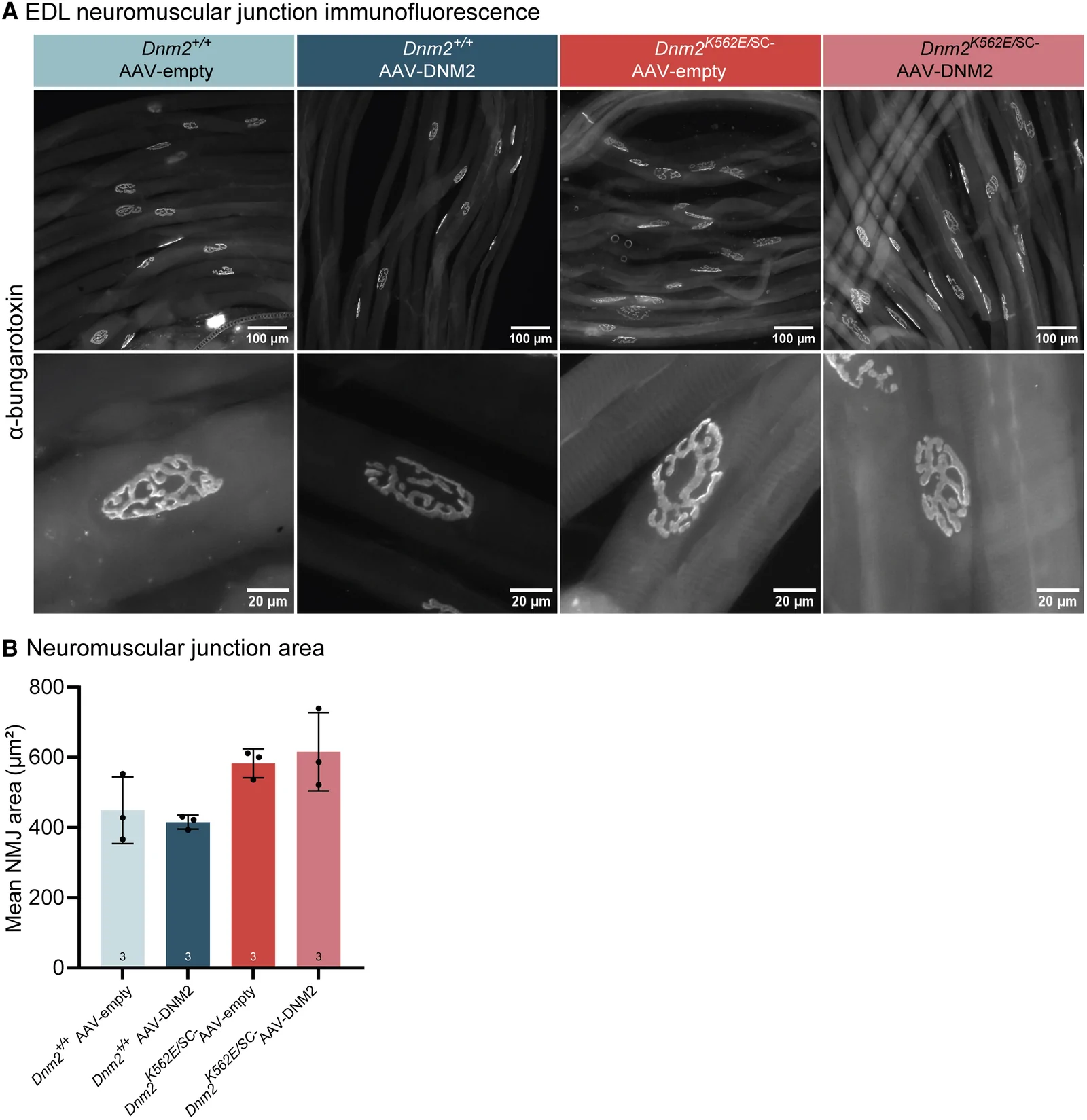
**Neuromuscular junction morphology in the *Dnm2^K562E/^*^SC*−*^ model.** (**A**) Fluorescent α-bungarotoxin labelling of isolated extensor digitorum longus (EDL) muscle fibre bundles. Upper panel, scale bar = 100 µm; lower panel, scale bar = 20 µm. (**B**) Mean neuromuscular junction (NMJ) area in EDL muscle. *n* = 3 mice per group. Ordinary one-way ANOVA (*F* = 4.928, *P* = 0.0317) with Tukey’s multiple comparisons. Each dot represents a mouse. Charts present individual values and mean ± standard deviation.

**Figure 10:**
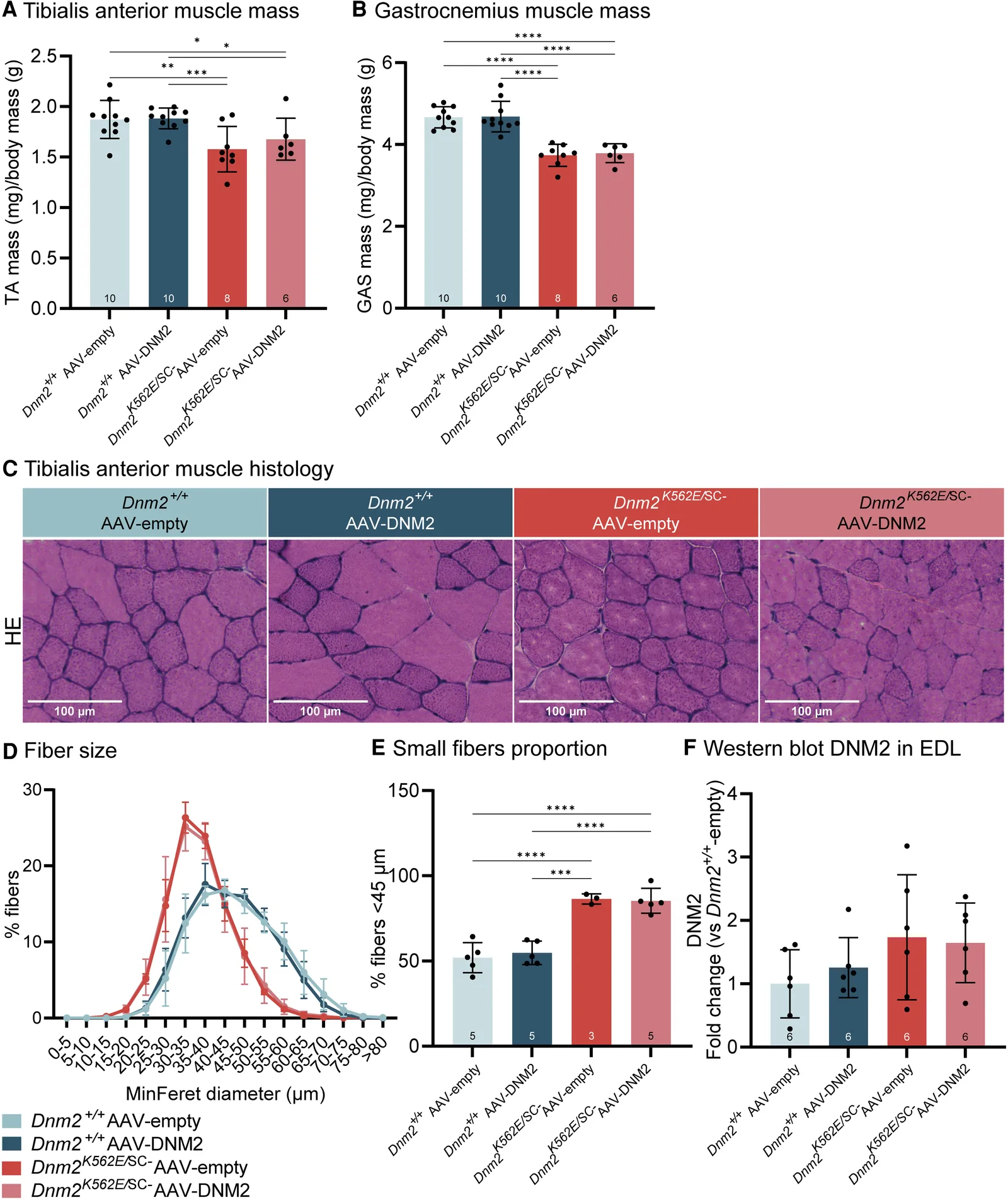
**The *Dnm2^K562E/^*^SC*−*^ model displays muscle atrophy that persists despite nerve-targeted DNM2 supplementation.** (**A–B**) Muscle mass of (**A**) Tibialis Anterior (TA) (ordinary one-way ANOVA (*F* = 6.210, *P* = 0.0021) with Tukey’s multiple comparisons) and (**B**) gastrocnemius (ordinary one-way ANOVA (*F* = 26.23, *P* < 0.0001) with Tukey’s multiple comparisons) normalized to body mass at 8 weeks. *Dnm2^+/+^*-empty, *n* = 10; *Dnm2^+/+^*-DNM2, *n* = 10; *Dnm2^K562E/^*^SC−^-empty, *n* = 8; *Dnm2^K562E/^*^SC−^-DNM2, *n* = 6. (**C**) TA transversal sections stained with haematoxylin-eosin (HE). *Dnm2^K562E^*^/SC−^ muscles display marked myofibre hypotrophy compared with controls. (**D**) TA fibres distribution based on their MinFeret diameter. (**E**) Proportion of small fibres (MinFeret<45 µm) in TA sections. Ordinary one-way ANOVA (F = 29.96, *P* < 0.0001) with Tukey’s multiple comparisons. In (**D**, **E**) *N*: *Dnm2^+/+^*-empty, *n* = 5; *Dnm2^+/+^*-DNM2, *n* = 5; *Dnm2^K562E/^*^SC−^-empty, *n* = 3; *Dnm2^K562E/^*^SC−^-DNM2, *n* = 5. (**F**) Quantification of DNM2 protein in EDL muscle, normalized to Ponceau S staining depicted in Supplementary material. *n* = 6 mice per group. Ordinary one-way ANOVA (*F* = 1.449, *P* = 0.2585) with Tukey’s multiple comparisons. Each dot represents a mouse. Charts present individual values and mean ± standard deviation, *P < 0.05, **P < 0.01, ***P < 0.001, ****P < 0.0001.

Since myelination deficits impair neural transmission from motor neurons, which can lead to neuromuscular junction (NMJ) disruption and secondary muscle pathology, we evaluated both NMJs and muscle tissue. NMJs integrity was assessed using α-bungarotoxin labelling of the extensor digitorum longus (EDL) muscle (Fig. 9A). No signs of fragmentation were revealed, but a non-significant trend towards increased NMJ area was observed in mutant groups compared to controls (Fig. 9B), potentially reflecting compensatory remodelling. Muscle mass was reduced in both the TA and gastrocnemius muscles of *Dnm2^K562E/^*^SC−^ mice (Fig. 10A–B). HE staining of TA sections revealed pronounced myofibre hypotrophy (Fig. 10C), confirmed by an increased proportion of small fibres (Fig. 10D–E). Western blot analysis showed no increase in DNM2 levels in the EDL muscle, which lies adjacent to the TA, confirming limited diffusion of the AAV from the intrathecal injection (Fig. 10F, Supplementary Fig. 9B). None of the muscle phenotypes were improved by Schwann cell-specific DNM2 overexpression. It is thus possible that part of the motor phenotype observed in this severe *DNM2*–CMT model arises from intrinsic muscle defects, as previously reported in the *Dnm2^K562E/+^* mouse.^42^

## Discussion

This study investigated the neuromuscular defects underlying CMT neuropathy caused by dominant negative loss-of-function mutations in *DNM2* and evaluated the therapeutic potential of increasing DNM2 expression.

Patients with *DNM2*–CMT experience distal motor and sensory deficits associated with markedly reduced nerve conduction velocity. Our detailed characterization of the *Dnm2^K562E/^*^SC−^ mouse validates this model as a robust tool for studying the peripheral nerve pathomechanism of *DNM2*–CMT (Supplementary Fig. 7). Although it is not genetically identical to patients, because *Dnm2* is selectively removed in Schwann cells in addition to the ubiquitous K562E mutation, its phenotype closely mimics the human condition and much more faithfully recapitulates neuropathic features than the *Dnm2^K562E/+^* mouse, which displays only mild symptoms and a predominantly myopathic component.^21^ The *Dnm2^K562E/^*^SC−^ model reveals a coherent cascade of pathological events that can explain patients’ phenotype. Reduced levels of the transcription factor EGR2 impair the activation of myelin genes, including MPZ, resulting in defective myelination characterized by decreased MPZ abundance and altered g-ratios. Impaired myelin disrupts axon-glia interactions and leads to a progressive loss of large-calibre myelinated axons, which are critical for rapid signal conduction. This axonal loss, combined with myelin thinning, produces the profound sensory and motor nerve conduction impairments observed in the model. Ultimately, these molecular and structural defects converge to produce the severe motor deficits characteristic of *DNM2*–CMT. These data also support an essential requirement for DNM2 during early postnatal Schwann cell maturation.

The rationale for supplementation with WT DNM2 in *DNM2*–CMT is a conceptually sound strategy. Indeed, CMT mutations in *DNM2* are loss-of-function.^16,17^ *In vivo*, complete loss of DNM2 in Schwann cells, whether constitutive or induced, leads to profound peripheral nerve defects,^18^ and expression of the K562E mutant allele alone is insufficient to sustain peripheral nerve integrity.^21^ Also, DNM2 reintroduction rescues the severe demyelination observed in Schwann cells lacking DNM2 *in vitro*.^43^ However, our *in vivo* findings indicate that post-symptomatic intervention is insufficient to reverse established neuropathy (Supplementary Fig. 7). Intrathecal DNM2 delivery at 4 weeks, a stage when motor deficits and severe demyelination are already present, failed to improve motor behaviour, nerve conduction or histological outcomes, despite robust transgene expression. This lack of efficacy suggests that the therapeutic window for effective DNM2 augmentation in Schwann cells may occur earlier, likely before or during the onset of active myelination, which begins around postnatal day 5 in mice.^44^ Consistent with this, previous work has shown that tamoxifen-induced DNM2 deletion in Schwann cells (*Dnm2*^SC−/SC−^) at 8 weeks causes demyelination that can be reversed by remyelination from non-recombined cells.^18^ In contrast, *Dnm2^K562E/^*^SC−^ mice lack normal DNM2 activity from early developmental stages, resulting in cumulative and potentially irreversible damage to myelin and axons. Therefore, our results may reflect both a missed developmental window and the presence of irreversible structural degeneration by the time of treatment. Alternative explanations include suboptimal promoter strength, AAV serotype or insufficient recovery period. In addition to timing considerations and in line with previous work,^20^ future studies could explore delivery of hyperactive gain-of-function DNM2 variants, which may overcome limitations of WT DNM2 supplementation after disease onset, and evaluate longer timepoints post-injection.

Overall, our results provide an in-depth characterization of a severe *DNM2*–CMT mouse model that closely recapitulates key electrophysiological and pathological features of the human neuropathy. This work establishes the essential role of DNM2 in peripheral nerve integrity and myelination and demonstrates that DNM2 supplementation after symptom onset is insufficient to reverse established pathology within the current experimental framework. The lack of therapeutic efficacy likely reflects a missed developmental window for effective intervention. These findings underscore the need for earlier or more sustained approaches and highlight that therapeutic strategies for *DNM2*–CMT may need to prevent, rather than repair, Schwann cell dysfunction. Together, these findings offer valuable guidance for refining future gene therapy strategies targeting *DNM2*-related CMT.

## Supplementary Material

fcag324_Supplementary_Data

## Data Availability

All source data supporting the findings of this study are provided with the article (Supplementary Table 3).
