# Supplementary material for: DNM2–Charcot–Marie–Tooth neuropathy stems from disrupted Schwann cell function and shows limited therapeutic reversibility
Source: Brain Commun. 2026 Sep 1;8(5):fcag324. doi: 10.1093/braincomms/fcag324 (PMC13594971; doi:10.1093/braincomms/fcag324)
Supplement: Supplementary file 1 [file fcag324_Supplementary_Data.zip › Headings_for_Supplementary_Tables_2_and_3.docx]

**Supplementary Table 2. Number of mice used per test and statistical analysis performed.** For each test, number total of mice and sex-repartition as well as statistical analysis.

**Supplementary Table 3. Source data underlying the main and supplementary figures.** Raw data used to generate the graphs and statistical analyses presented in the manuscript.
