# Supplementary material for: DNM2–Charcot–Marie–Tooth neuropathy stems from disrupted Schwann cell function and shows limited therapeutic reversibility
Source: Brain Commun. 2026 Sep 1;8(5):fcag324. doi: 10.1093/braincomms/fcag324 (PMC13594971; doi:10.1093/braincomms/fcag324)
Supplement: Supplementary file 1 [file fcag324_Supplementary_Data.zip › Supplementary_material.pdf]

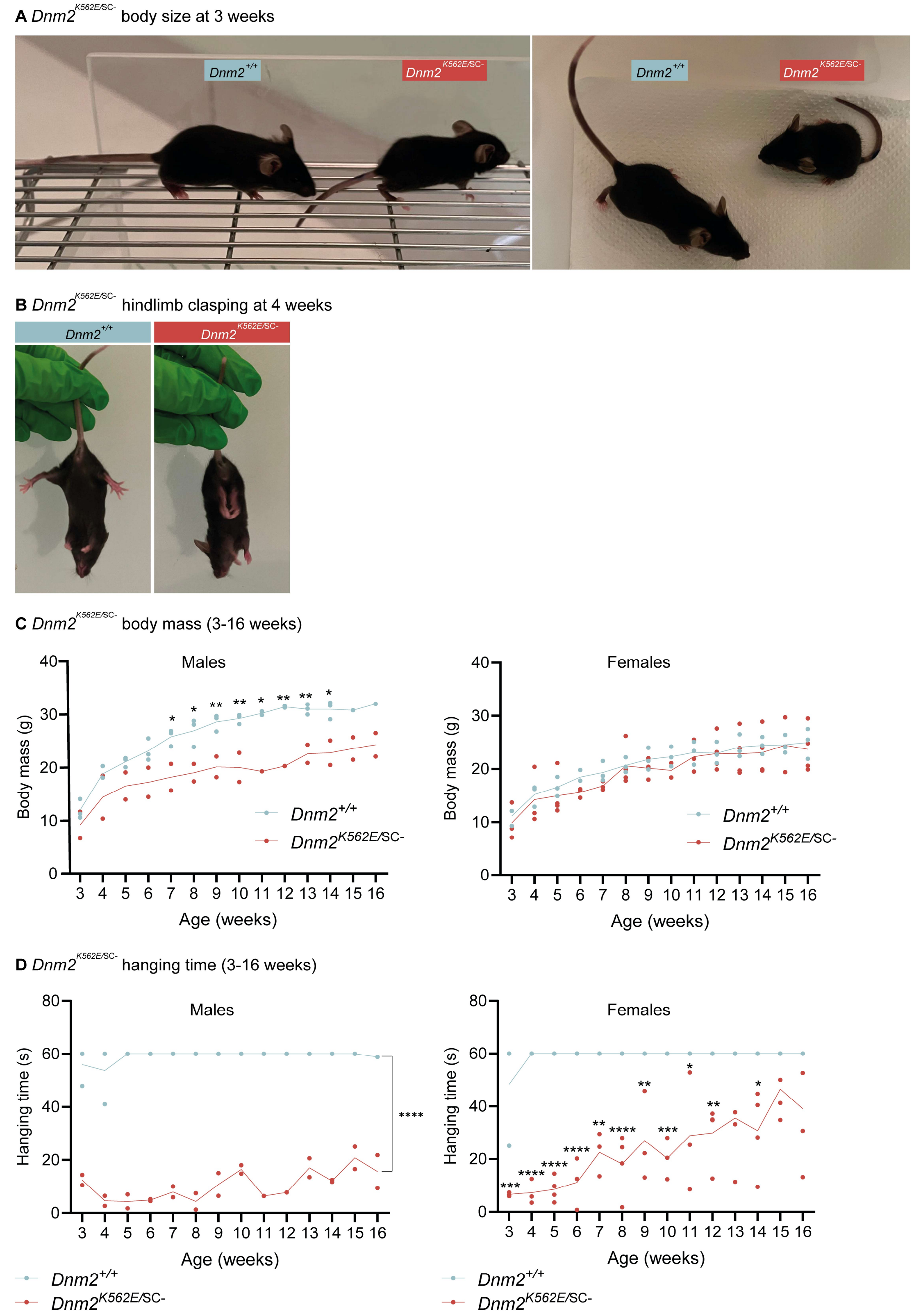

Supplementary Fig 1. General characterization of the *Dnm2*$^{K562E/SC-}$ mouse line. (A) Representative images of *Dnm2*$^{+/+}$ control and *Dnm2*$^{K562E/SC-}$ mice at 3 weeks. **(B)** Tail suspension at 4 weeks, indicating presence or absence of hindlimb clasping. **(C)** Body mass from 3 to 16 weeks. Males: *Dnm2*$^{+/+}$, n=3; *Dnm2*$^{K562E/SC-}$, n=2. Two-way ANOVA (interaction F=0.9447, P=5208, age F= 17.34, P<0.0001, genotype F= 127.6, P<0.0001) with Bonferroni's multiple comparisons. Females: *Dnm2*$^{+/+}$, n=3; *Dnm2*$^{K562E/SC-}$, n=4. Two-way ANOVA (interaction F=0.1337, P=0.9998, age F= 11.91, P<0.0001, genotype F= 4.007, P=0.0498) with Bonferroni's multiple comparisons. **(D)** Hanging time from 3 to 16 weeks. Males: *Dnm2*$^{+/+}$, n=3; *Dnm2*$^{K562E/SC-}$, n=2. Two-way ANOVA (interaction F=1.755, P=0.0934, age F= 2.226, P=0.0307, genotype F= 1926, P<0.0001) with Bonferroni's multiple comparisons. Females: *Dnm2*$^{+/+}$, n=3; *Dnm2*$^{K562E/SC-}$, n=4. Two-way ANOVA (interaction F=1.872, P=0.0510, age F= 2.674, P=0.0048, genotype F= 238.5, P<0.0001) with Bonferroni's multiple comparisons. Each datapoint represents one mouse; lines represent group means, *p<0.05, **p<0.01, ****p<0.0001.

Therapeutic strategy

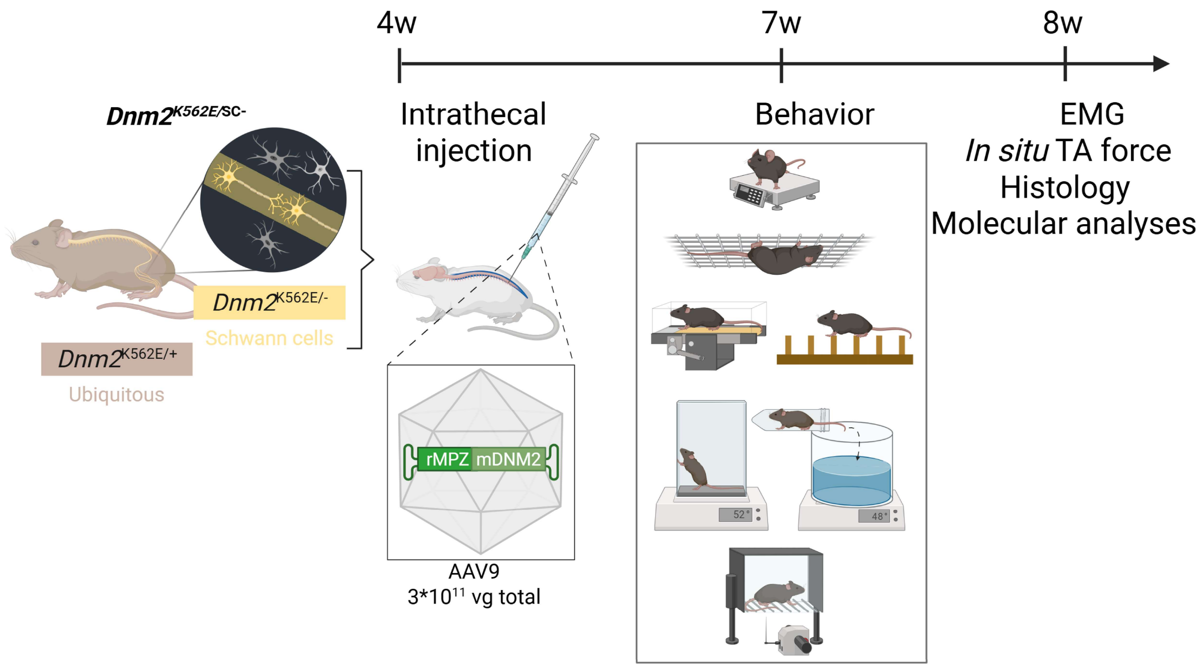

Supplementary Fig 2. Experimental set-up. *Dnm2*$^{K562E/SC-}$ mice, harboring the heterozygous K562E CMT mutation ubiquitously and a Schwann cell-specific deletion of the second *Dnm2* allele, were injected intrathecally at 4 weeks of age (after disease onset) with AAV9 carrying murine *Dnm2* under the control of the Schwann cell-specific MPZ promoter. Behavioral analyses were performed at 7 weeks of age, and functional analyses and tissue collection at 8 weeks of age. TA= Tibialis Anterior. EMG= electromyography. Created in BioRender. Goret, M. (2026). https://BioRender.com/9k6l9cg.

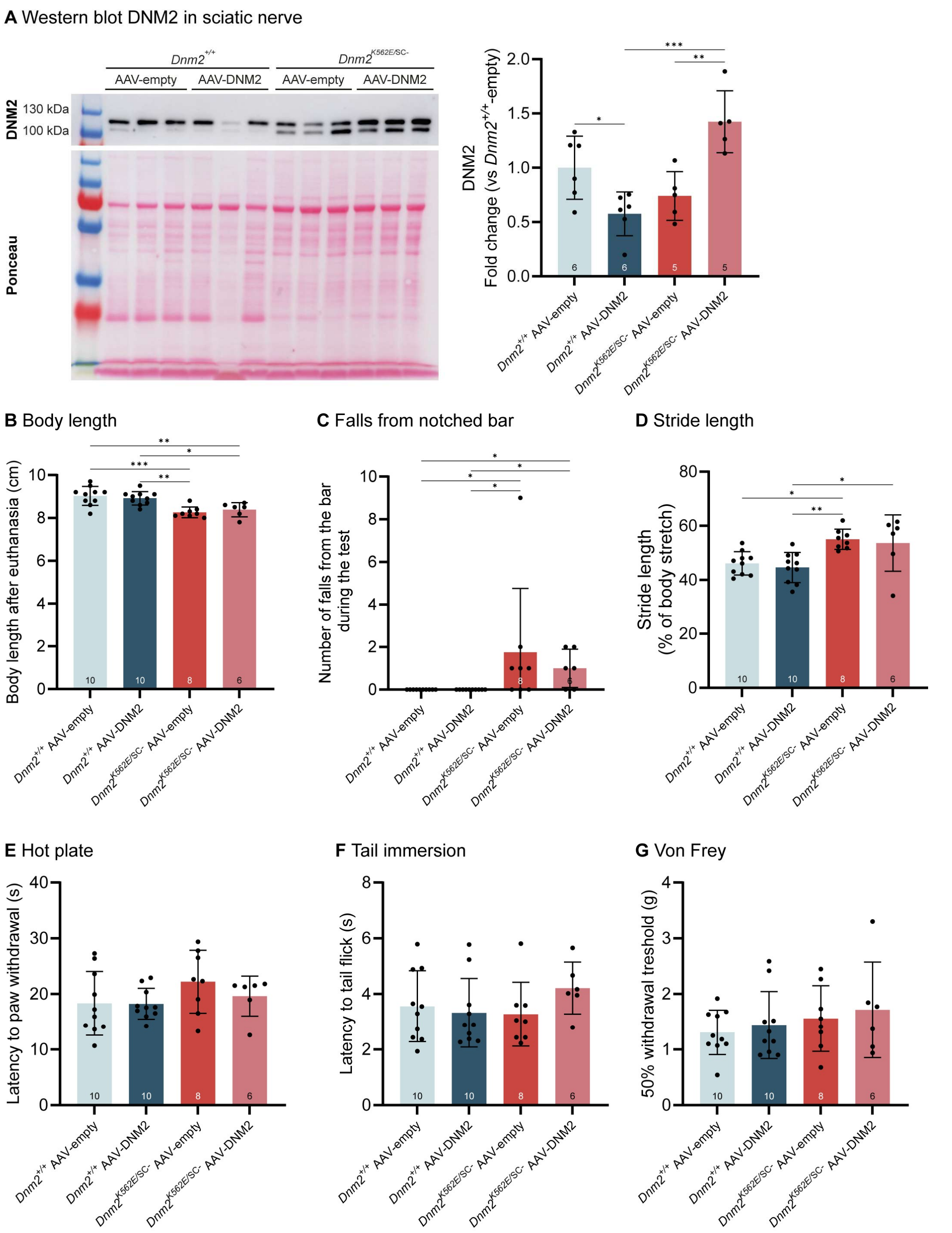

Supplementary Fig 3. Validation of DNM2 overexpression in nerves and additional behavioral assessments. (A) Representative western blot and quantification of DNM2 protein level in sciatic nerve at 8w, normalized to Ponceau S staining. *Dnm2*$^{+/+}$-empty, n=6; *Dnm2*$^{+/+}$-DNM2, n= 6; *Dnm2*$^{K562E/\mathrm{SC-}}$-empty, n=5; *Dnm2*$^{K562E/\mathrm{SC-}}$-DNM2, n= 5. Ordinary one-way ANOVA (F=11.33, P=0.0002) with Tukey's multiple comparisons. Uncropped images of this blot are shown in Supplementary Material. **(B)** Body length (nose to tail base) measured after euthanasia at 8w. Ordinary one-way ANOVA (F=10.28, P<0.0001) with Tukey's multiple comparisons. **(C)** Number of falls from the notched bar during the 10-trials test at 7w. Kruskal-Wallis (P=0.0009) with Dunn's multiple comparisons. **(D)** Stride (=length of a step) during treadmill walking normalized to body stretch at 7w. Ordinary one-way ANOVA (F=6.420, P=0.0017) with Tukey's multiple comparisons. **(E)** Hot plate test (52°C) at 7w. Reaction latency is recorded. Kruskal-Wallis (P=0.3408) with Dunn's multiple comparisons. **(F)** Tail immersion test (48°C) at 7w. Reaction latency is recorded. Ordinary one-way ANOVA (F=1.085, P=0.3706) with Tukey's multiple comparisons. **(G)** Von Frey test at 7w. Reaction to mechanic stimuli is recorded. The data presents the gram value at which the mouse is predicted to react 50% of the time. Ordinary one-way ANOVA (F=0.4963, P=0.6876) with Tukey's multiple comparisons. In (B-G) N: *Dnm2*$^{+/+}$-empty, n=10; *Dnm2*$^{+/+}$-DNM2, n= 10; *Dnm2*$^{K562E/\mathrm{SC-}}$-empty, n=8; *Dnm2*$^{K562E/\mathrm{SC-}}$-DNM2, n= 6. Each dot represents a mouse. Charts present individual values and mean ± standard deviation, *p<0.05, **p<0.01, ***p<0.001.

A Scatter plot of g-ratio as a function of axon diameter

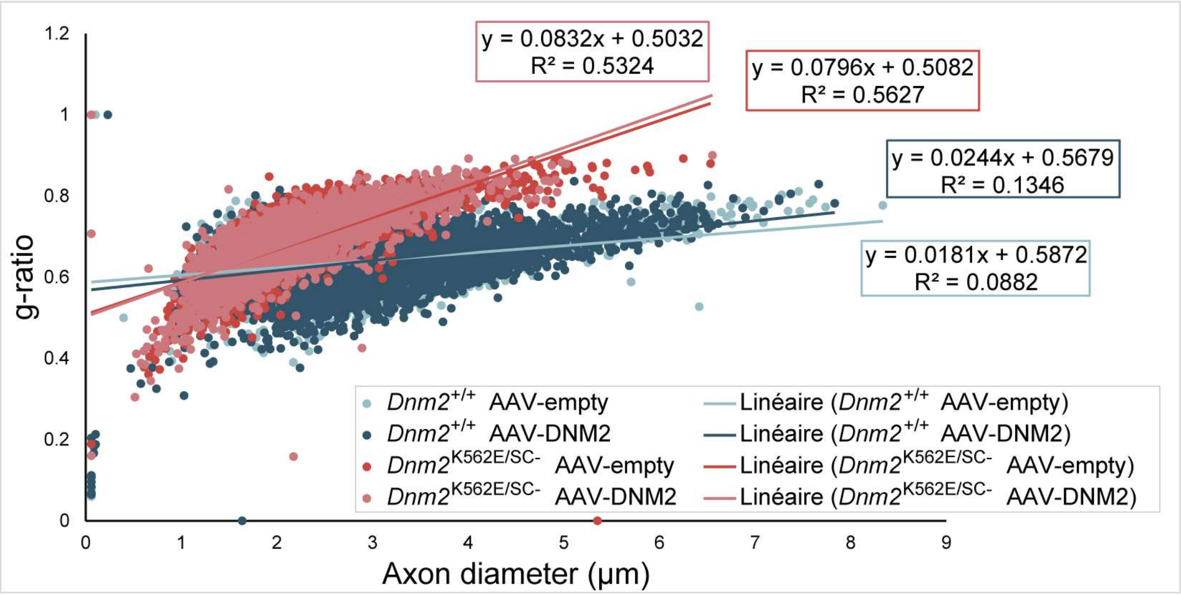

B Scatter plot of myelin thickness as a function of axon diameter

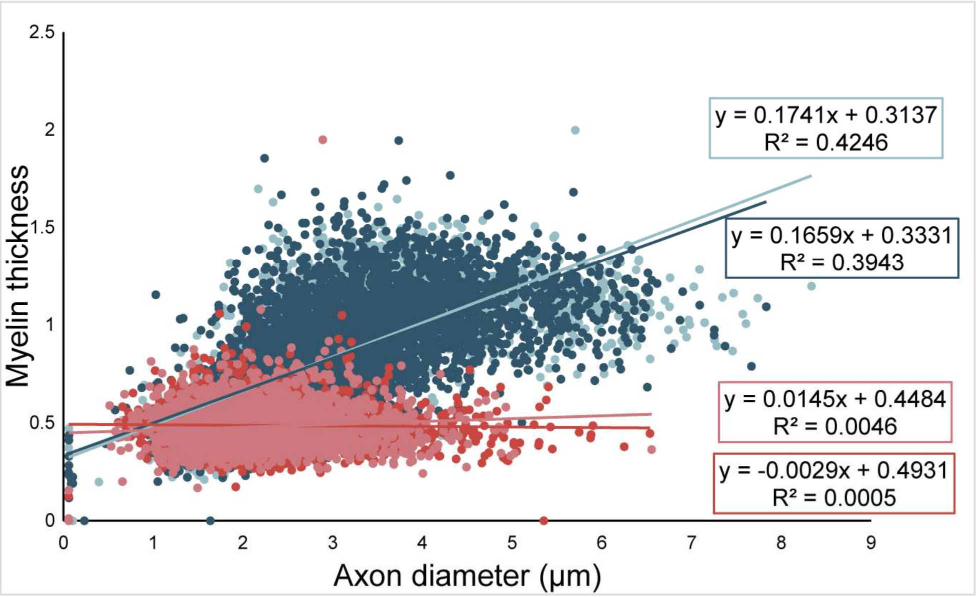

Supplementary Fig 4. Relationship between axon diameter, g-ratio, and myelin thickness. (A-B) Scatter plots of **(A)** g-ratio, and **(B)** myelin thickness as a function of axon diameter. Linear regression lines with R^2 and slope are shown for descriptive purposes only to illustrate overall trends among non-independent datapoints; no statistical comparisons were performed. *Dnm2*$^{+/+}$-empty, n=3 mice, 3447 fibers; *Dnm2*$^{+/+}$-DNM2, n= 3 mice, 4560 fibers; *Dnm2*$^{K562E/SC-}$-empty, n=3 mice, 2496 fibers; *Dnm2*$^{K562E/SC-}$-DNM2, n=3 mice, 2398 fibers; single fibers are plotted.

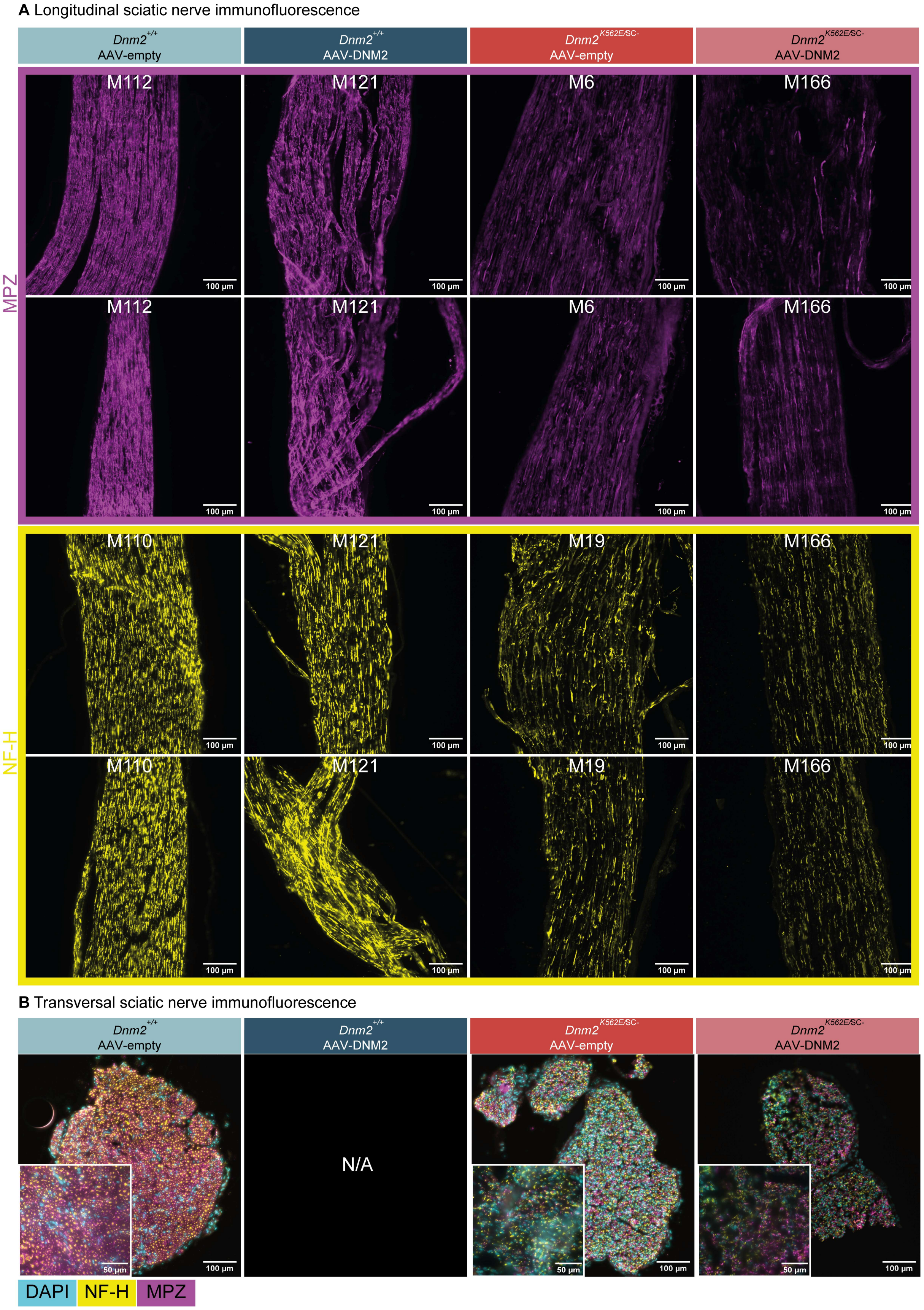

Supplementary Fig 5. Regional consistency of MPZ and NF-H alterations in *Dnm2*$^{K562E/SC-}$ sciatic nerves. **(A)** Longitudinal sciatic nerve sections immunolabeled for Myelin Protein Zero (MPZ) (upper panels, magenta outlines) and Neurofilament Heavy (NF-H) (lower panels, yellow outlines) at two distinct anatomical locations within the same animal, showing comparable staining patterns across regions. Scale bar= 100µm. **(B)** Transversal mid-sciatic nerve sections immunolabeled for MPZ and NF-H, confirming findings observed in longitudinal sections. Main images acquired at 20× magnification (scale bar = 100 µm); insets (lower left corner) at 63× magnification (scale bar = 50 µm). M=male.

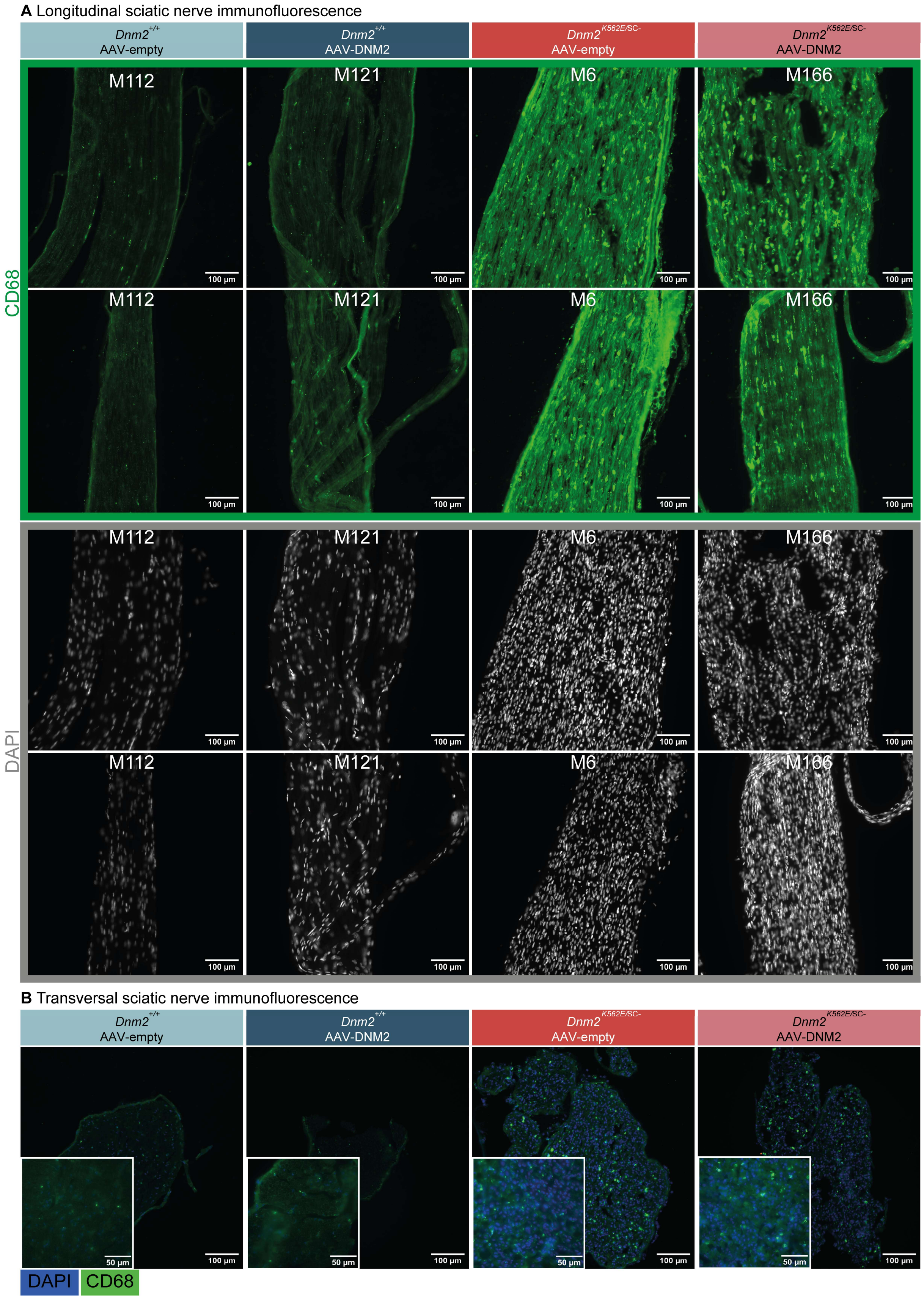

Supplementary Fig 6. Regional consistency of immune cells infiltration in *Dnm2*$^{K562E/SC-}$ sciatic nerves. (A) Longitudinal sciatic nerve sections immunolabeled for CD68 (upper panels, green outlines) and DAPI (lower panels, gray outlines) at two distinct anatomical locations within the same animal, showing comparable staining patterns across regions. Scale bar= 100µm. **(B)** Transversal mid-sciatic nerve sections immunolabeled for CD68 and DAPI, confirming findings observed in longitudinal sections. Main images acquired at 20× magnification (scale bar = 100 µm); insets (lower left corner) at 63× magnification (scale bar = 50 µm). M=male.

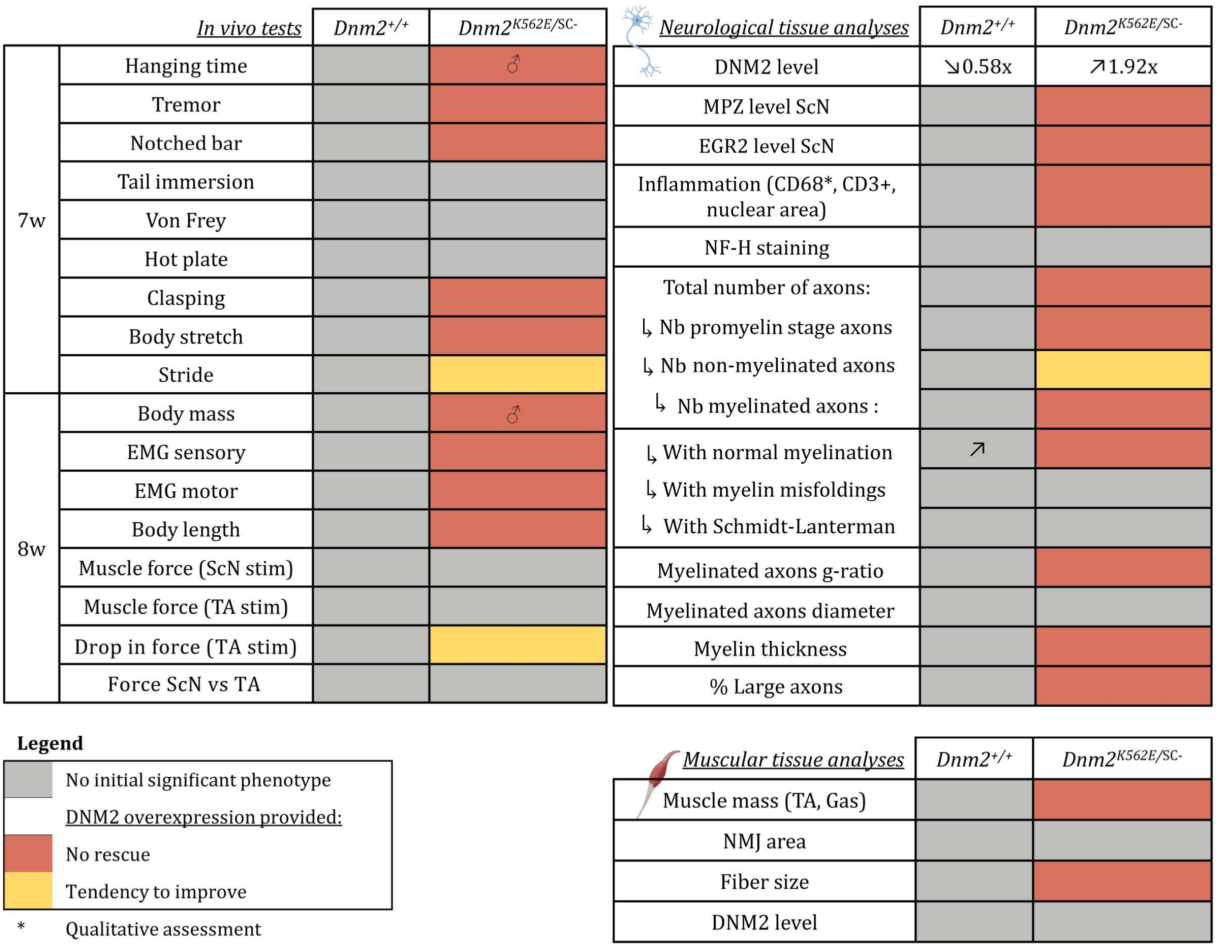

Supplementary Fig 7. Overview of outcomes following DNM2 overexpression in *Dnm2$^{K562E/SC-}$* mice. Summary of outcomes after intrathecal injection of murine DNM2 at 4w in *Dnm2$^{+/+}$* and *Dnm2$^{K562E/SC-}$* mice. Assessments were performed at 7-8w using in vivo tests and tissues analyses. TA= Tibialis anterior. ScN= sciatic nerve. Gas= Gastrocnemius. NMJ= neuromuscular junction. Tendency to improve= treated mutants are not different from both untreated mutants and controls.

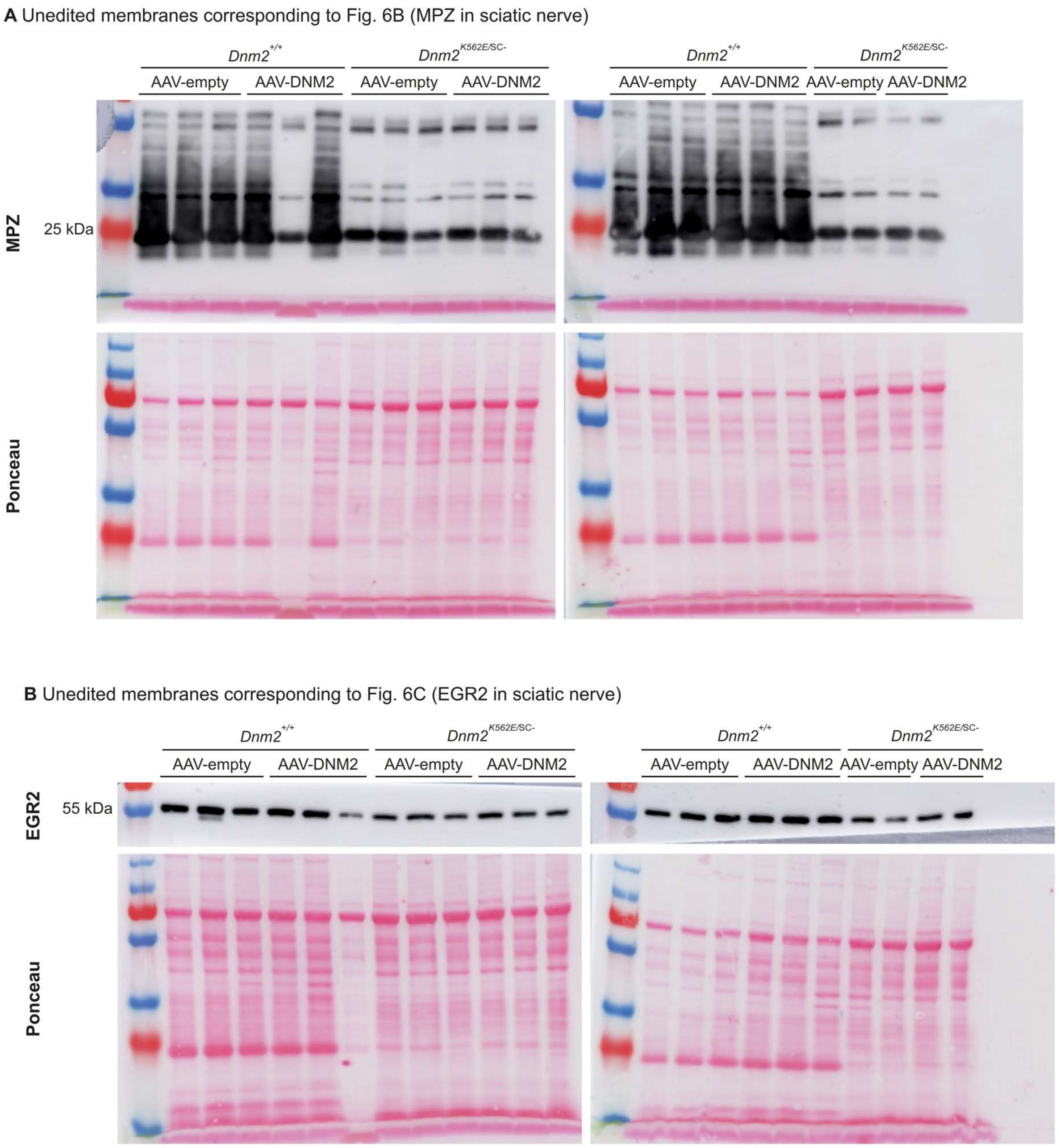

Supplementary Fig 8. Uncropped and unprocessed western blot membranes for Fig. 6B and 6C. (A) Membrane corresponding to Fig. 6B and **(B)** membrane corresponding to Fig. 6C. Protein ladders and Ponceau S staining are shown.

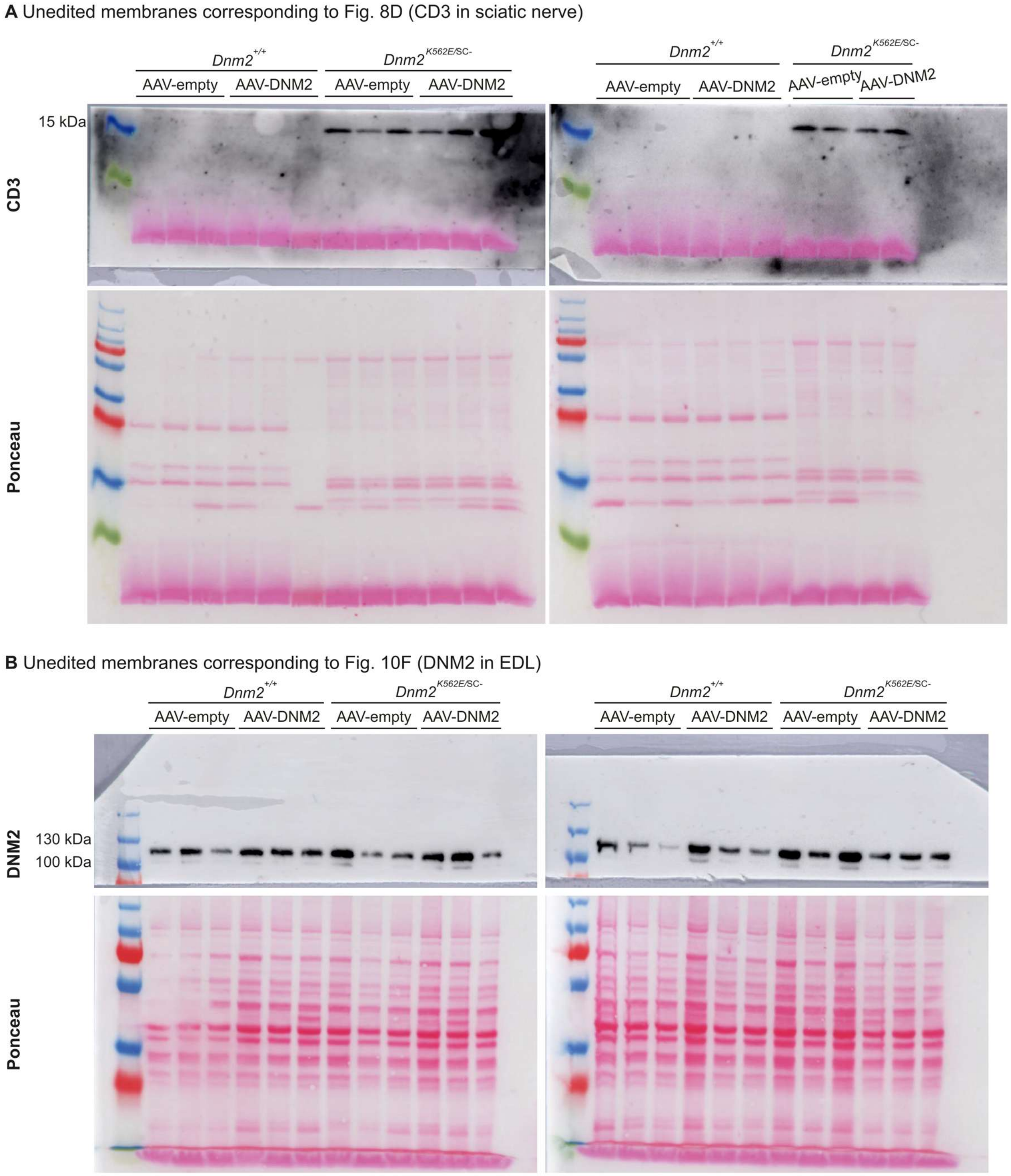

Supplementary Fig 9. Uncropped and unprocessed western blot membranes for Fig. 8D and 10F. (A) Membrane corresponding to Fig. 8D and **(B)** membrane corresponding to Fig. 10F. Protein ladders and Ponceau S staining are shown.

Unedited membranes corresponding to Supplementary Fig. 3A (DNM2 in sciatic nerve)

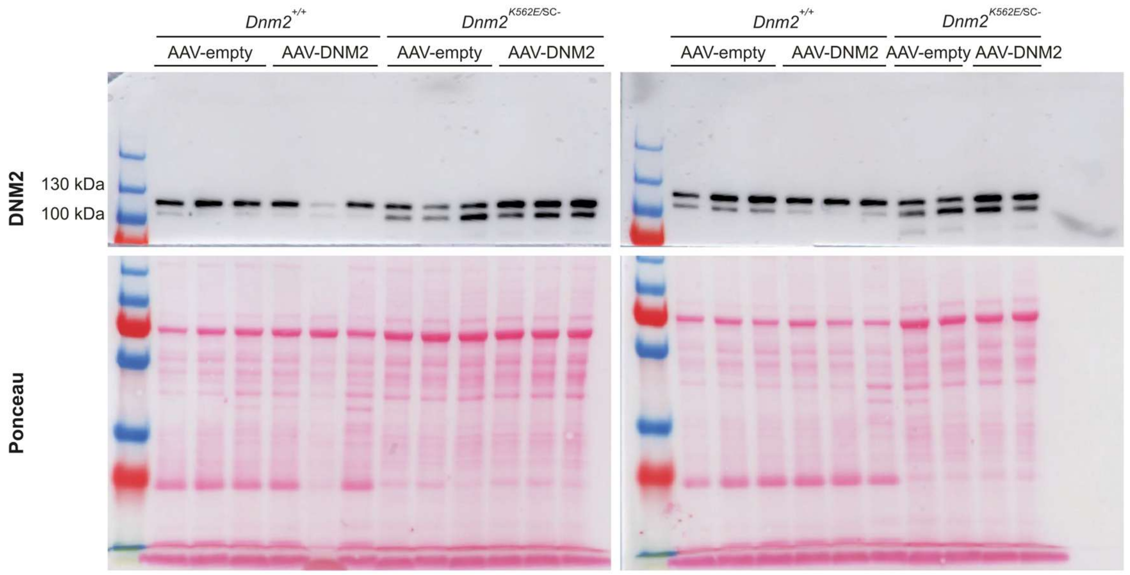

Supplementary Fig 10. Uncropped and unprocessed western blot membranes for Supplementary Fig. 3A. Protein ladder and Ponceau S staining are shown.

Reagent/Resource	Reference or Source	Identifier or Catalog Number
**Muscle immunofluorescence antibodies (dilution)**		
CF488A Alpha-Bungarotoxin (1:1000)	INTERCHIM SA	00005
**Sciatic nerve immunofluorescence antibodies (dilution)**		
MPZ, rabbit polyclonal (1:200)	Abcam	31851
↳ GAR Alexa 555, goat polyclonal (1:250)	Invitrogen	A21430
CD68, rat monoclonal (1:200)	Bio Rad	MCA 1957GA
↳ GARat Alexa 488, goat polyclonal (1:250)	Thermo Scientific	A-11006
DAPI (1:1000)	/	/
NFH, chicken polyclonal (1:500)	Abcam	4680
↳ GAC Alexa 488, goat polyclonal (1:250)	Invitrogen	A11039
**Western blot antibodies (dilution)**		
DNM2, rabbit polyclonal (1:1000)	Homemade (2865)	N/A
MPZ, rabbit polyclonal (1:1000)	Abcam	31851
EGR2, rabbit monoclonal (1:1000)	Abcam	108399
CD3, mouse monoclonal (1:1000)	Homemade	N/A
GAM perox, goat polyclonal (1:10000)	Jackson Immunoresearch	115-036-068
GAR perox, goat polyclonal (1:10000)	Jackson Immunoresearch	111-036-045
**Genotyping PCR oligos**	5'-sequence-3'	
6115 Er KE	TACACTGTCTGCACTGTCGAGCCCTG	
6116 Ef KE	GCCATCTTCAACACAGAGCAGAGGTG	
Cre 160	GAACCTGATGGACATGTTCAGG	
Cre 161	AGTGCGTTCGAACGCTAGAGCCTGT	
4613	GGCAGCAAGCCTGTTTACCCGC	
4614	GCTGAGCCTCACAGTGCAGAGCC	
**AAV titration qPCR oligos**		
mDNM2 Fw	ACCCCACACTTGCAGAAAAC	
mDNM2 Rv	CGCTTCTCAAAGTCCACTCC	
CMVe For	TACGGTAAACTGCCCACTTG	
CMVe Rev	AGGAAAGTCCCATAAGGTCA	

Supplementary Table 1. Reagents used. Antibodies used for immunofluorescences and western blots. Primers used for PCR and qRT-PCR.
